# Processing difficulties and biochemical barriers in camel milk fermentation

**DOI:** 10.3389/fmicb.2026.1752671

**Published:** 2026-02-02

**Authors:** Sifatun Nesa Ali, Mutamed Ayyash, Afaf Kamal-Eldin

**Affiliations:** 1 Department of Food Science, College of Agriculture and Veterinary Medicine, United Arab Emirates University, Al Ain, United Arab Emirates; 2 National Water and Energy Center, United Arab Emirates University, Al Ain, United Arab Emirates

**Keywords:** bovine milk yogurt, camel milk yogurt, fermentation, microstructure, starter cultures, texture

## Abstract

Camel milk (CM) is recognized for its high nutritional enrichment, and the distinction from bovine milk mainly because of its unique protein composition, proteolytic products, and anti-microbial compounds. These unique chemical properties have positive effects on the nutritional value but negative impact on the sensory attributes and consumer acceptability of fermented CM. This review summarizes the current state of knowledge on CM fermentation, emphasizing the influence of milk composition on gel and microstructure formation, texture, and overall quality of the products. While the richness of fermented CM in bioactive peptides enhances its nutritional and therapeutic values, major challenges are associated with their thin consistency and weak gel structure. Various strategies to overcome these challenges and develop unique functional fermented CM products are discussed, including the use of alternative starter cultures (e.g., *Lactobacillus helveticus, Lb. casei*, and *Lactiplantibacillus plantarum*), stabilizing additives hydrocolloids or proteins, as well as optimized heat treatments, high-pressure processing and other emerging technologies. Despite several processing and formulation procedures, no particular approach has yet offered a comprehensive solution for achieving firm and stable camel yogurt. Therefore, it is important to accept fermented CM products as being different and develop means to improve their sensory quality and consumer acceptance. Overall, this review underscores the necessity for ongoing research to optimize the quality and commercial viability of fermented CM.

## 1 Introduction

**Figure 1:**
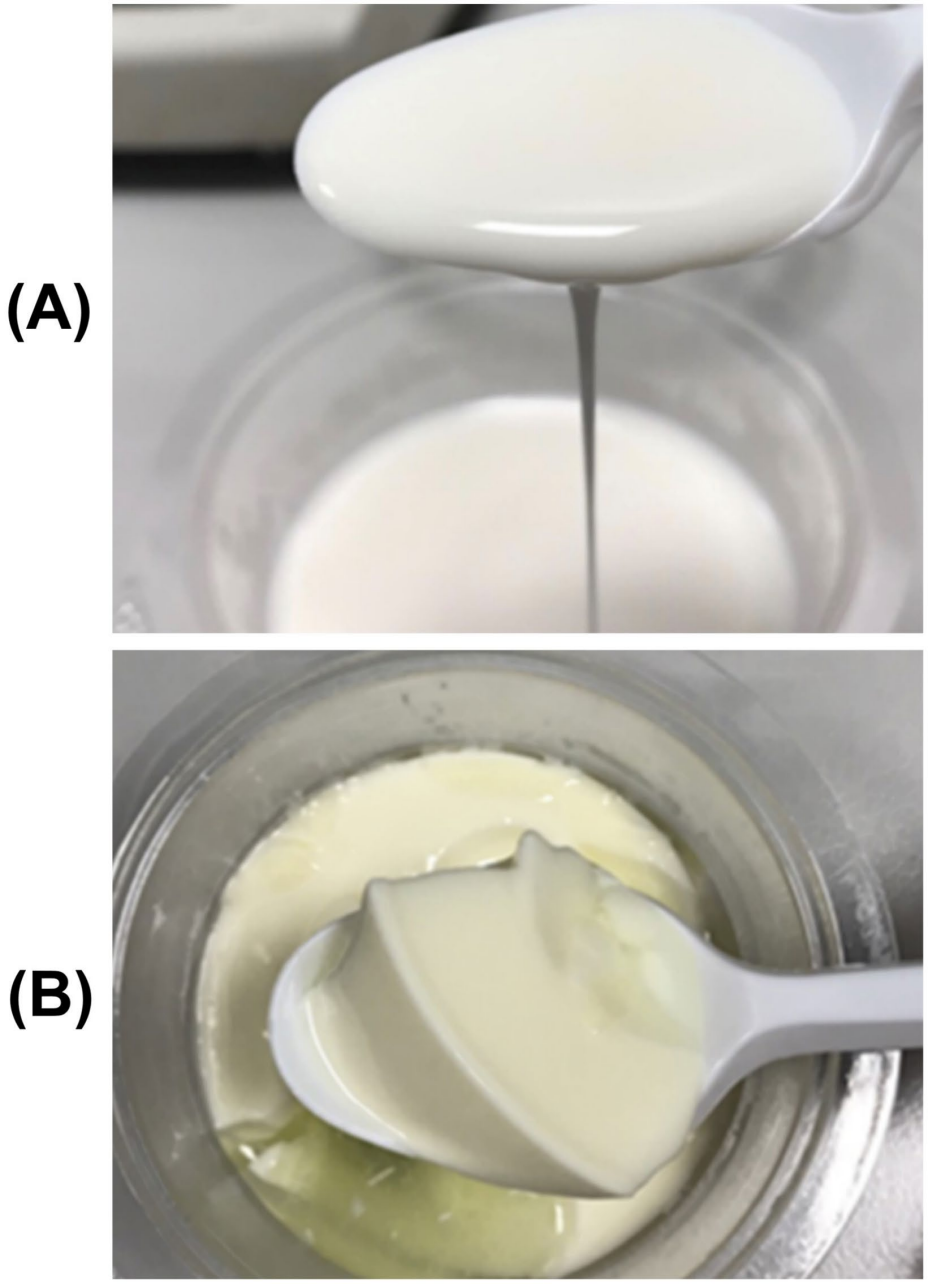
Differences in appearance and consistency of fermented milk: **(A)** Fermented camel milk exhibiting a thin, pourable consistency with limited gel strength. **(B)** Bovine yogurt showing a firm, cohesive, and strong gel [reproduced from Sobti and Kamal-Eldin (2019) under creative commons permission].

The Middle East and North Africa along with some regions in Asia are major consumers of camel milk (CM) (Muthukumaran et al., 2023). The worldwide camel population is estimated to be 32.7 million, with approximately 87.1% located in the Middle East and North Africa region (Oselu et al., 2022a). Recently, CM has attracted broader global interest, including in Europe and North America, owing to its potential health benefits, including antidiabetic, anticancer, and hypoallergic properties (Mullaicharam, 2014; Benmeziane-Derradji, 2021; Mohamed et al., 2021; Swelum et al., 2021; Kamal-Eldin et al., 2021; Ho et al., 2022a; Alia et al., 2023). However, unlike bovine milk (BM), CM consistently generates think and liquid-like fermented products (Figure 1). In particular, consumers typically opt for yogurts with dense texture and well-balanced viscous behavior but compared to BM yogurt, fermented camel milk is characterized by much lower hardness (52.5 vs. 11.8 g) and viscosity (661 vs. 0.57 Pa.sec) (Sobti and Kamal-Eldin, 2019). The microstructural properties, texture, and rheology of fermented dairy products, specifically yogurt and cheese, are of critical importance to their quality as primary determinants of appearance, mouthfeel, and overall consumer acceptability (Alia et al., 2023). Thus, CM is more suitable for the manufacture of fermented drinkable product compared to BM yogurts (Sobti et al., 2021; Sobti et al., 2023).

These differences between fermented CM and BM products have been attributed to several factors, including its unique chemical composition, complex colloidal system, protein micelle and fat globule sizes, and antimicrobial compounds (Mohamed et al., 2021; Mohamed et al., 2020; Mohamed et al., 2022a; Mbye et al., 2022; Arain et al., 2023a; Hamed et al., 2024). Comparison of the microstructures of CM and BM-acid gels revealed a denser casein network in BM than in CM because the smaller size of the casein micelles in the latter result in greater bonding during acidification (Glantz et al., 2010). The relatively low level of *κ*-casein, lack of *β*-lactoglobulin, high β-casein content, and large casein micelles are considered to contribute to the poor coagulation leading to the weak structure, and thin consistency of fermented CM products compared to fermented BM products (Ho et al., 2022a; O’Kennedy et al., 2006).

The formation of fermented CM may be facilitated with the use of certain additives such as citrate that can dissociate the micelles into monomers, leading to improvement to the texture of the CM gel (Wang J. et al., 2025). The nutritional value and consumer perception of these products can also be enhanced by adding additional proteins (Lesme et al., 2020), hydrocolloids (Saleh et al., 2018), and/or fruit pulp (Sobti et al., 2023). In addition, new technologies, optimization of processing and fermentation conditions, and the use of stabilizers or fortifications could help overcome these challenges (Arain et al., 2023b). Although extensive studies have been conducted on creating various fermented BM products with flavorings, colorants, and sweeteners; however, similar investigations on fermented CM products remain limited (Sobti et al., 2023).

Several reviews have emphasized the hypoallergenic, anticarcinogenic, and antimicrobial properties of CM, along with its potential application in the development of functional and probiotic foods (Hamed et al., 2024; Ansari et al., 2024; Almasri et al., 2024; Marete et al., 2024; Seyiti et al., 2024). In addition to the health-promoting aspects, the global status, production trends, and economic value chain of the CM industry have been detailed, identifying both market opportunities and operational challenges (Ait El Alia et al., 2025). The high nutritional and therapeutic value of fermented CM products has generated great interest in understanding the reasons contributing to their weak gels and watery consistency in order to find appropriate strategies to overcome these challenges (Benmeziane-Derradji, 2021; Mohamed et al., 2021; Swelum et al., 2021; Ho et al., 2022a; Alia et al., 2023). The aim of this review is to synthesize and extend this current knowledge by focusing on the properties of fermented CM products and how they are affected by the chemical composition of the milk, fermentation bacteria, processing conditions, and stabilizing agents. This knowledge is essential for the further design of strategies that will facilitate the production of nutritional and therapeutic fermented CM products with improved consumer acceptance.

## 2 Chemical and structural transformations during fermentation

The yogurt fermentation process is preceded by heat treatment to facilitate the formation of complex networks between the soluble whey proteins and *κ*-casein through thiol/disulfide bond interchanges (Asaduzzaman et al., 2021). Heating milk prior to fermentation affects the degree of whey protein denaturation and the formation of micelle-bound and soluble complexes with *κ*-casein (Anema, 2021). The micelle-bound complexes are responsible for increasing the final firmness of acid gels, while the soluble complexes enhance the water-holding capacity and firmness of yogurt gels (Asaduzzaman et al., 2021). However, the contributions of the abundance and chemical constitution of denatured whey protein/*κ*-casein complexes to the gelation processes and the textural properties of the heated milk and final products remain unclear (Donato et al., 2007). Nevertheless, it is known that the milk type along with the heating and fermentation treatments themselves significantly impact the properties of yogurt gels, mainly the microstructure, firmness, rheology, water-holding capacity, and whey separation (Mahomud et al., 2017).

Heating of BM above 70 °C cause denaturation of *β*-lactoglobulin, the major whey protein in the milk, thereby exposing its free thiol (-SH) group (Mahomud et al., 2021). The thiol/disulfide exchange reactions between the free thiol group (-SH) of denatured β-lactoglobulin and the S-S bonds of *κ*-casein in BM are well-documented (Asaduzzaman et al., 2021; Mahomud et al., 2017). The formation of intermolecular S-S bridges through covalent bonds and hydrophobic interactions occurring at temperatures below 75 °C depends on the *β*-lactoglobulin concentration (Mahomud et al., 2017). These intermolecular thiol/disulfide interchange reactions during heating and subsequent acidification result in covalent bonds that improve the microstructure (Gazi and Huppertz, 2015) and enhance the strength of yogurt gels (Mahomud et al., 2021). Unlike *β*-lactoglobulin, *α*-lactalbumin, as the major whey protein in CM, has no free thiol group and therefore cannot initiate thiol/disulfide exchange reactions. However, these reactions can be initiated by camel serum albumin or other whey proteins, enabling *α*-lactalbumin to cross-link with the micellar caseins in CM. Another difference between α-lactalbumin and *β*-lactoglobulin is that the former readily forms a molten globule, an intermediate between the native and completely denatured form of the protein, under even mild denaturation conditions (Permyakov, 2020). The low concentration of *κ*-casein in CM is another factor that may affect the formation and concentration of the whey protein/κ-casein complex.

**Figure 2:**
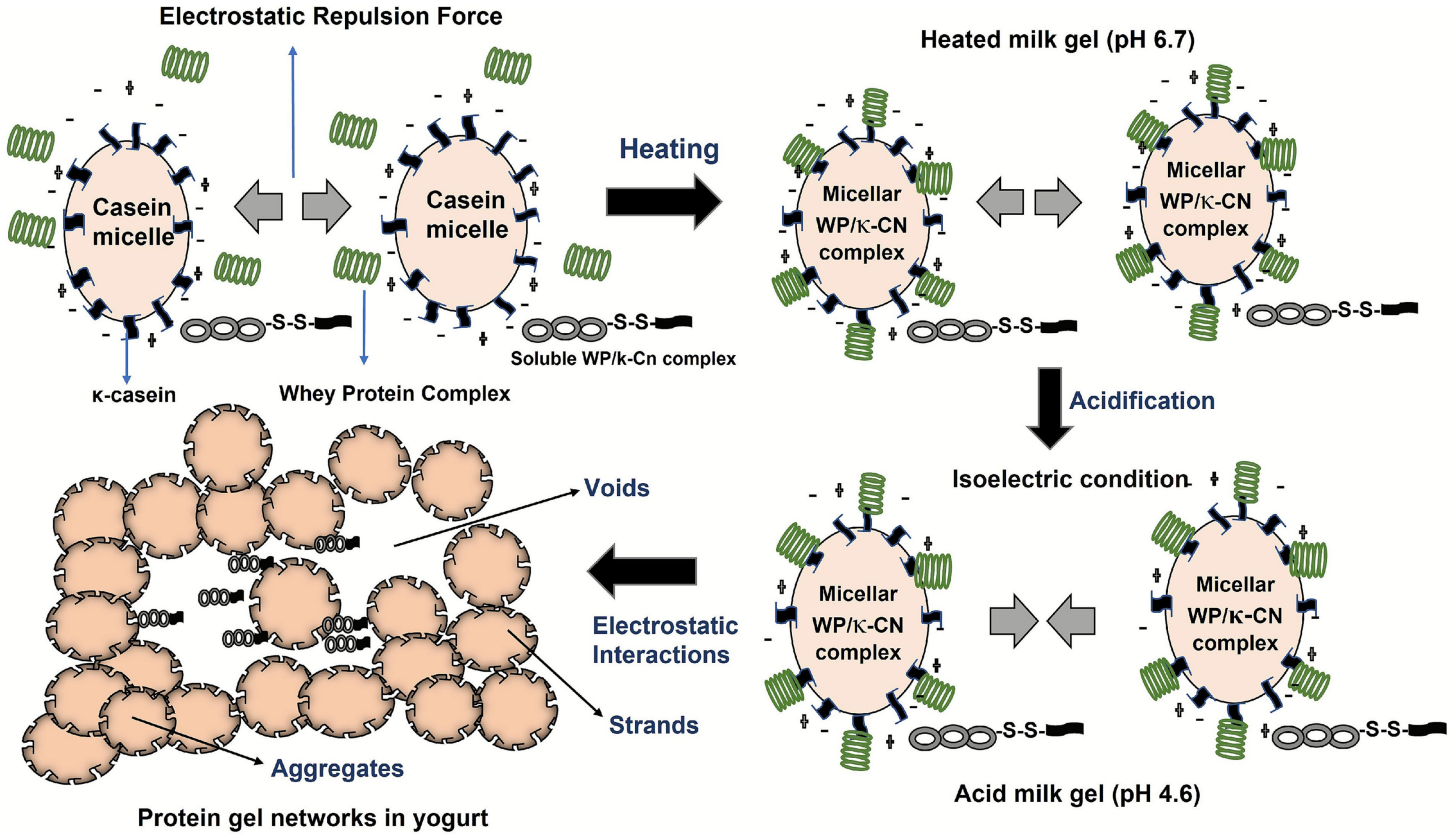
Schematic representation of the protein network formed in yogurt gels by the pre-fermentation heat treatment and acidification by bacterial cultures. Heating causes whey protein (WP) to denature and to form soluble and micelle-bound WP–*κ*-casein (WP/κ-CN) complexes via thiol–disulfide (–S–S–) interactions. Subsequent acidification to the isoelectric point of milk reduces electrostatic repulsion between the casein micelles and promote their aggregation, resulting in the formation of a protein gel network characterized by interconnected aggregates, strands, and voids typical of yogurt structure [adapted from Asaduzzaman et al. (2021) and Mahomud et al. (2017)].

Bacterial fermentation gradually reduces the milk pH. Once the pH reaches the isoelectric point of caseins (pH 4.3 for CM and pH 4.6 for BM), the casein micelles are destabilized owing to a decrease in their net negative charges, electrostatic repulsions, and steric stabilization (Meletharayil et al., 2015). As shown in Figure 2, this destabilization leads to the coagulation of caseins and the formation of three-dimensional gel networks, influenced by the milk protein complexes (Arab et al., 2023). The -SH/S-S interchange reactions in the micelle-bound whey proteins continue to form during acidification (pH < ~6.7), leading to an increase in the gel’s storage modulus (G′) (Mahomud et al., 2017) and the final elastic properties of the yogurt (Zhao et al., 2021). The soluble whey protein/*κ*-casein complexes also play a critical role in the structural characteristics of acid gels (Mahomud et al., 2017; Lakemond and van Vliet, 2008; Chever et al., 2014). During acidification, some casein micelles may disintegrate due to the solubilization of colloidal calcium phosphate (Sinaga et al., 2016). This process decreases the overall negative charge and electrostatic repulsion while enhancing hydrophobic attractions between micelles. Consequently, the storage modulus (G′) of the yogurt elevates, encouraging protein aggregation, linkage formation, and expansion of the protein network (Nguyen et al., 2018).

**Figure 3:**
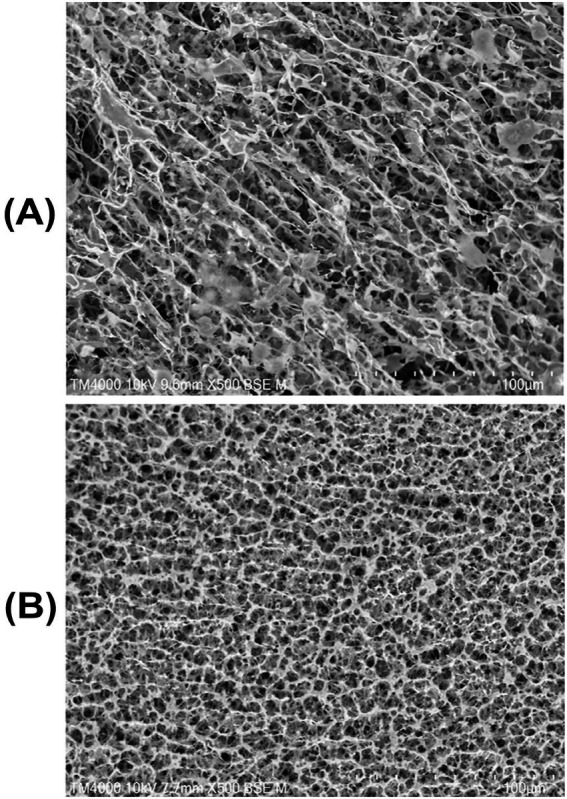
Differences in the microstructures of yogurt gels formed from caseins: **(A)** Fermented camel milk gel exhibiting a loose, heterogenous network characterized by elongated protein strands and large voids, and **(B)** fermented bovine milk gel displaying a compact, homogenous protein network with smaller pores and higher structural continuity. The fermented camel milk is characterized by elongated strands and large voids, while the bovine milk provides a denser structure [reproduced from Wang et al. (2025) under creative commons permission].

The microstructure of yogurt is based on a casein matrix consisting of strands, aggregates, and voids (Figure 3) (Prasanna et al., 2018). Fermentation transforms milk from a Newtonian fluid to a semi-solid gel, in which the created protein network retains liquid in the voids or pores (Arab et al., 2023). Polymerized whey proteins and whey protein– *κ*-casein complexes fill these voids, leading to more compact microstructures in BM (Bierzuńska et al., 2019; Fang and Guo, 2019). However, in the fermented CM hydrogel, these voids can retain water, resulting in less syneresis (whey separation) compared to that occurring in BM under the same conditions. The network responsible for water retention or expulsion is determined by the cross-linked protein matrix and clusters of casein micelles with distinct globular forms, as well as the polysaccharides and fat globules contained within the yogurt’s microstructure (Arab et al., 2023; Kim et al., 2020). Syneresis occurs in bovine yogurt as a result of physicochemical changes that cause gel shrinkage, signifying the release of bound whey from the continuous network (Lee and Lucey, 2010). Syneresis is regulated through two groups of factors: (1) factors influencing water-holding capacity, including physical network density, formation, stability, and chemical water elimination; and (2) factors enhancing water removal from networks, including intra-network stresses (rearrangement, fast cooling, acidification) and extra-network stresses (Arab et al., 2023). Understanding yogurt microstructures can therefore help to determine the degree of compact cluster formation and syneresis (Sobti et al., 2020). For a yogurt to resist syneresis, it is essential to maintain appropriate gel stiffness and water-holding capacity (Gilbert et al., 2020). Bovine yogurt typically has smaller voids due to the highly aggregated protein clusters, whereas camel yogurt has larger voids in its microstructure with great water-holding capacity (Sobti et al., 2020). Owing to the absence of gel stiffness, stirred yogurt can also undergo less syneresis compared to set yogurt (Arab et al., 2023). Water retention in the voids makes fermented CM softer, more elastic, and more liquid (Ho et al., 2022a; O’Kennedy et al., 2006). Despite the lower degree of syneresis in camel than bovine yogurt, the shrinkage of the gel structure renders the camel gel more susceptible to delayed and increased syneresis during storage or handling (Oselu et al., 2022b). Indeed, the syneresis index of BM yogurt decreases considerably during storage when CM is added, indicating an increase in the water-holding capacity (Kamal-Eldin et al., 2020).

Exopolysaccharides (EPS), produced during bacterial fermentation of milk, play crucial roles in yogurt quality by influencing the gelling, stabilizing, thickening, and water-binding abilities; preventing syneresis; and improving texture without compromising sensorial attributes (Mende et al., 2016). EPS also exhibit functional properties such as antioxidant activity, prebiotic benefits, and immunomodulatory effects (Riaz Rajoka et al., 2020). Charged EPS bind to protein networks, while uncharged EPS accumulate in the serum, causing thermodynamic incompatibilities (Laneuville and Turgeon, 2014). EPS affect the stability of yogurt by interacting with caseins, whey proteins, bacterial cells, and minerals, affecting complexation, co-solubility, or incompatibility (Mende et al., 2016). Negatively charged EPS induce repulsive interactions, whereas neutral EPS promote phase separation (Han et al., 2023). Yogurt with EPS-producing strains has higher consistency but lower yield stress, elastic modulus, and viscosity due to the EPS forming channels that decrease protein interactions and prevent syneresis (Hassan et al., 2003). *Lactobacillus delbrueckii* ssp*. bulgaricus* and *Streptococcus thermophilus* are key EPS producers, which are known to enhance a BM yogurt’s viscosity and sensory attributes (Tiwari et al., 2021) through cross-feeding and promoting metabolites exchange (Liu et al., 2016).

**Figure 4:**
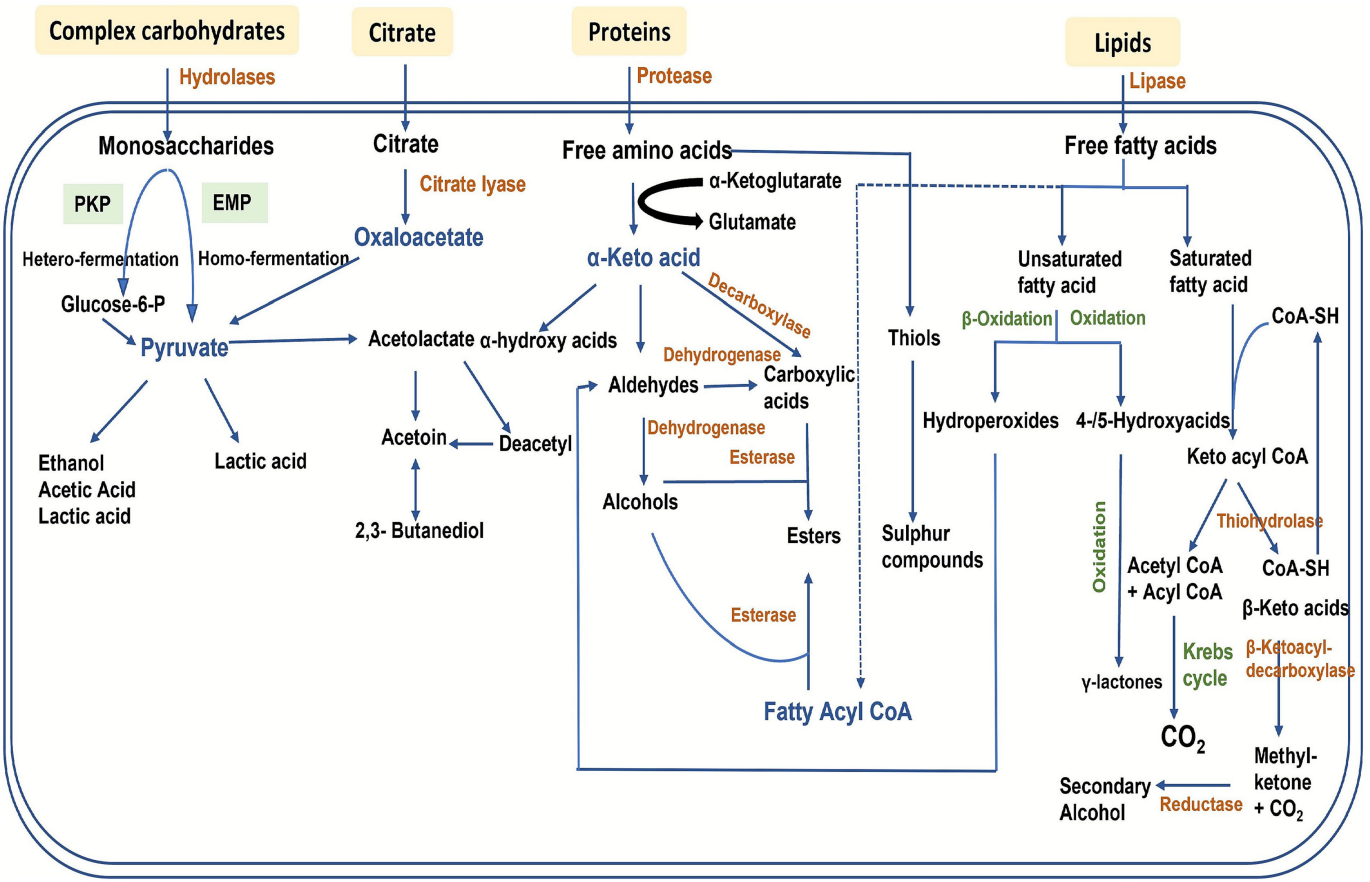
Overview of general metabolic pathways used by lactic acid bacteria (LAB) during milk fermentation. Carbohydrate, protein, and lipid metabolism by LAB leads to the production of key flavor compounds, including organic acids, alcohols, aldehydes, ketones, and sulphur containing compounds [adapted from Hu et al. (2022)] with permission from Taylor & Francis.

During fermentation, lactic acid bacteria (LAB) release enzymes that metabolize milk components through glycolysis, proteolysis, and lipolysis, producing lactic acid, other organic acids, peptides, and free amino and fatty acids, which influence the yogurt’s flavor and texture (Figure 4) (Smid and Kleerebezem, 2014; Bintsis, 2018; Hayek and Ibrahim, 2013). Along with glycolysis and lipolysis, proteolysis is a crucial process in the production and development of fermented dairy products given its major impact on texture and flavor. The generated amino acids and peptides function as precursors for other catabolic processes that produce both desirable and undesirable flavors and odors (Shori, 2017), playing major roles in the bioactivities of fermented products. Proteolysis, caused by *Lactobacillus* spp., affects the texture and aroma of yogurt made from fermented CM and negatively affects the rheological parameters during storage (Gandhi and Shah, 2014). Yogurts may shear thin when there is an increase in total solids due to elevated proteolytic activity (Abd El-Aziz et al., 2022; Costa et al., 2022). Differences in buffering capacity, proteolytic activity, and antimicrobial proteins contribute to observed variations in acidity between the fermented products prepared from CM and BM (Sobti et al., 2021). The proteolytic systems of LAB significantly impact protein, peptide, and amino acid availability for growth and the final rheological and sensory properties of fermented foods (Hayek and Ibrahim, 2013). Caseins are the primary substrates for LAB proteolytic systems during fermentation and storage, as evidenced by reduced electrophoretic band intensities for *κ*-, *β*-, and *α*-caseins (Li et al., 2019), while the whey proteins α-lactalbumin and β-lactoglobulin remain largely unaffected (González-Olivares et al., 2014). By producing essential growth factors such as peptides and amino acids, proteolysis may have an influence on the ability of probiotics to survive and thrive in fermented dairy products (Tavakoli et al., 2019; Chelladhurai et al., 2024). LAB strains such *Lactiplantibacillus plantarum* influence the proteolysis of milk when co-fermented with *S. thermophilus* during cold storage (Li et al., 2019), and these strains also impact the structure, nutritional profile, and functional properties of both fermented CM and BM (Imen et al., 2015). Use of a combination of *Lactobacillus helveticus* and *L. plantarum* strains improved BM protein degradation and decreased protein antigenicity (Zhao et al., 2021).

## 3 Key factors affecting camel yogurt quality

### 3.1 Milk composition and characteristics

Table 1 compares the chemical composition of CM and BM and shows major differences in the relative casein proportions and micelle properties. CM is very different from BM, including the paler (whitish) color, slightly salty flavor, and lower density (1.029 g/mL) (Izadi et al., 2019). The distinct composition of CM results in a relatively weaker response to traditional gel production methods used with BM, such lactic acid fermentation or renin treatments (Attia et al., 2001). Because texture has a major impact on sensory and quality parameters such as mouth feel, appearance, and customer acceptance, the poor coagulation properties of CM render commercial fermented CM products, including yogurt and cheese, with generally low acceptance by consumers (Patel et al., 2022). Although stabilizers, skim milk powder, calcium chloride, and commercial yogurt culture have been used in a series of initiatives to produce high-quality yogurt from CM, the texture and consistency of the gels remain undesirable (Al-Zoreky and Al-Otaibi, 2015). Another disadvantage is that the fermentation of CM generally takes longer than required for BM fermentation (Galeboe et al., 2018). Therefore, further research is needed to optimize the operating parameters and standardize procedures to improve the production process and acceptability of fermented CM (Seifu, 2023).

**Table 1:** Chemical composition of proteins, fats, lipids in camel and bovine milk.

Components	Camel milk	Bovine milk	Reference
Protein (%)	3.1	3.4	Mohamed et al. (2020)
Total caseins content (%)	50–88	80–84	Mohamed et al. (2020) and Fabiano et al. (2013)
αS1-, *α*S2-, β-, κ-caseins (relative %)	26:4:67:3	38:10:36:12	Mohamed et al. (2020)
Casein micelle’s diameter size (nm)	20–300	40–160	Park and Haenlein (2013)
Whey proteins (WP) (%)	20–25	18–20	Mohamed et al. (2020) and Fabiano et al. (2013)
β-lactoglobulin (% of WP)	-	53.6	Tsermoula et al. (2021), Tsermoula et al. (2021), Mohamed et al. (2022a), Hailu et al. (2016), and Hailu et al. (2016)
α-lactalbumin (% of WP)	27	20.1	
Lactoferrin (μg/mL)	639–210	76.7–140	
Serum albumin (%)	26	6.2	
Immunoglobulins A, G, & M (g/l)	1.5 (18%)	0.3 (5.3%)	
Fat (%)	2.9–5.4	3.7–4.4	Singh et al. (2017) and Imamou Hassani et al. (2022)
Dry matter of cholesterol (mg/100 g)	37.15	25.63	Konuspayeva and Faye (2021)
Triacylglycerols (%)	96	>98%	Ereifej et al. (2011)
Branched-chain fatty acids (%)	3.03	1.82	Dreiucker and Vetter (2011)
Fat globule’s diameter size (μm)	3.2–5.6	4.3–8.4	Khalesi et al. (2017)
Lactose (%)	4.5	4.7	Mohamed et al. (2021)

In a previous study, conducted by our research team, showed that the properties of fermented CM and BM are affected by the milk type, bacteria, temperature, and their interactions (Chelladhurai et al., 2024). Compared to BM, CM is void of *β*-lactoglobulin, has lower *κ*-casein and higher β-casein contents, has larger casein micelles, smaller fat globules, and higher levels of proteolytic products (Mohamed et al., 2020; Mbye et al., 2022; Chelladhurai et al., 2024; Tsermoula et al., 2021). As discussed above (Figure 2), *β*-lactoglobulin and *κ*-casein play a very important role in yogurt quality. The size of casein micelles in CM (20–300 nm) is two-folds larger than that of the micelles in BM (40–160 nm) (Swelum et al., 2021), which may potentially influence the final texture and consistency of fermented CM. Differences in protein profiles and amino acid sequences between CM and BM can also significantly influence the composition of their fermented products (Izadi et al., 2019). The relative composition of *α*S1-, αS2-, *β*-, and *κ*-caseins in CM was found to be 26:4:67:3 compared to a ratio of 38:10:36:12 in BM (Mohamed et al., 2020). This difference results in higher concentrations of β-casein and lower levels of α-caseins in CM. Furthermore, CM lacks β-lactoglobulin and has lower levels of κ-casein (3.5%) than BM (10%), which are the two most important proteins for the initial formation of yogurt gels (Mohamed et al., 2022a). Moreover, CM exhibits a higher content of whey proteins (20–25%) than BM (18–20%) (Mohamed et al., 2020; Fabiano et al., 2013). Together, these variations contribute to differences in the ultimate rheological properties of fermented CM, such as weaker coagulation and gel structure, from those of BM yogurt (Hailu et al., 2016).

**Figure 5:**
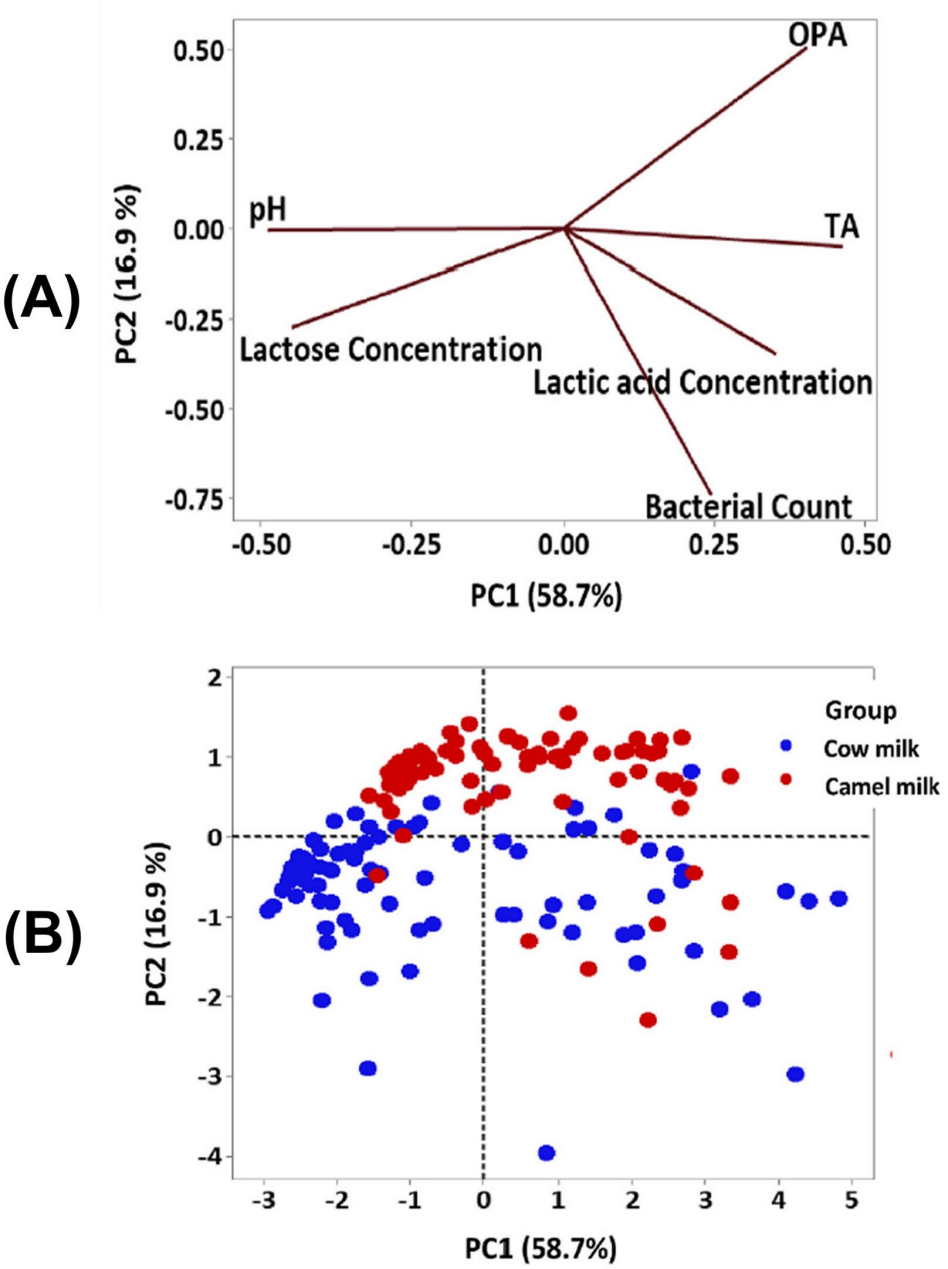
Principal component analysis showing the effect of milk type on the chemical properties of fermented camel and bovine milk. The upper panel **(A)** shows the loading plot (TA = titratable acidity; OPA = proteolytic products) and the lower panel **(B)** shows the score plot for camel and bovine milk [reproduced from Chelladhurai et al. (2024) with permission from Elsevier].

A crucial factor that affects the fermentability of CM and BM is the degree of proteolysis of the milk proteins by the bacteria and indigenous factors (Chelladhurai et al., 2024). CM shows a higher degree of proteolysis than BM, which correlates negatively to the bacterial count (Figure 5). In addition, *β*-casein, which is highly abundant in CM, is more susceptible to proteolysis than the other caseins. CM contains shorter β-casein chains with more proline residues, and its hydrolysis produces bioactive peptides and releases antioxidative amino acids such as phenylalanine and tryptophan (Izadi et al., 2019). Such an increase in proteolytic products enhances the water holding capacity and the watery consistency of Fermented CM and influences the gel’s internal stability (Abdeldaiem et al., 2022).

CM exhibits antimicrobial activities that may affect bacterial growth during fermentation (Swelum et al., 2021; Hamed et al., 2024). CM significantly surpasses BM in serum albumin content (26% vs. 6.2%) (Mbye et al., 2022) and in the contents of immunoglobulins A, G, and M (18% *vs* 3.5%) (Hailu et al., 2016). Moreover, CM is significantly richer in lactoferrin and lysozyme compared to BM (639.4–2094.9 μg/mL *vs* 76.7–140 μg/mL), making it more potent in immune defense and antimicrobial activities (El-Agamy, 2000; Kappeler et al., 1998). The lactoferrin in CM has been shown to exhibit antibacterial, antiviral, anticancer, anti-inflammatory, and immunomodulatory effects. Similarly, CM contains higher levels of lysozyme, in the range of 23.3–71.4 μg/mL compared to only 7 μg/mL lactoferrin found in BM (Elagamy et al., 1996). This elevated lysozyme concentration enhances CM’s antibacterial properties, further contributing to its potential health benefits (Mohamed et al., 2022a). These notable differences in protein composition might contribute to a denser texture of CM compared to BM.

In casein micelles, calcium binds to αS1-, αS2-, and *β*-caseins in the core, while *κ*-casein forms a “brush” on the micelle surface (de Kruif and Zhulina, 1996; Dalgleish and Corredig, 2012). CM caseins are characterized by lower degree of phosphorylation and micelle stability compared to those of BM (Sobti and Kamal-Eldin, 2019; Ryskaliyeva et al., 2018; Kumar et al., 2015). The higher levels of β-casein and their greater chaperone activity in CM compared to BM (Kumar et al., 2015) can further increase the amphiphilicity and detergent-like properties of CM proteins, which impairs their aggregation and refolding (Raynes et al., 2015). In addition, the structure and properties of fermented CM were improved by adding bovine casein and whey proteins (Sobti and Kamal-Eldin, 2019).

The functional properties of fermented CM may also be affected by the milk lipids. Compared to BM, CM exhibits a slightly higher fat content (Table 1) and smaller fat globule size (Khalesi et al., 2017), which may be linked to the slower and more thorough creaming process (Lean, 2011). In particular, CM has a higher concentration of branched-chain fatty acids (3.03%) than BM (1.82%) (Dreiucker and Vetter, 2011) and also has a markedly lower carotene content (Claeys et al., 2014). These differences result in a “waxy texture” and whiter color of fermented CM (Ereifej et al., 2011), possibly influencing its digestibility and sensory attributes (Tomotake et al., 2006). The CM fat composition is dominated by higher-molecular-weight triacylglycerols (≥C40 carbons), with low levels of C24–C40 and high levels of C48–C52, resulting in a higher melting temperature (32.6 °C) compared to that of BM (22.8 °C) (Smiddy et al., 2012). Goat milk (GM) yogurt exhibits higher water-holding capacity than BM yogurt due to its higher protein (3.27% vs. 3.14%) and fat (3.69% vs. 3.33%) contents (Ragab et al., 2021). Although understanding the role of fat is essential for developing CM products with desired characteristics, the effects of fat content on the textural and rheological properties of fermented CM remain to be thoroughly investigated.

### 3.2 Role of starter cultures and fermenting bacteria

CM producers worldwide have developed diverse traditional fermented products, each with its own microbiological, physicochemical, and flavor characteristics (Table 2). These products vary according to fermentation conditions and microbial properties, which are then reflected in the unique sensory characteristics (Hamed et al., 2024; Shori, 2017; Seifu, 2023). Compared to fermented BM yogurts, the majority of fermented CM products are beverages (i.e., drinkable yogurts) (Sobti et al., 2021; Sobti et al., 2023; Kamal-Eldin et al., 2020). For example, Chal, a slightly thick beverage popular in Bulgaria, Iran, and Turkey, is fermented by a complicated bacterial consortium mainly consisting of *L. plantarum*, *Lactobacillus kefiri*, and *Enterococcus faecium* (Hamed et al., 2024; Seifu, 2023; Soleymanzadeh et al., 2016). Doogh, which is consumed in Iran, Afghanistan, and Turkey, is a drinkable yogurt fermented with *Bifidobacterium bifidum* and frequently enriched with salt, ginger extract, pectin, or gum Arabic (Azarikia and Abbasi, 2010). Gariss, which is produced by spontaneous fermentation in Sudan, has a liquid to slightly thick consistency (Hamed et al., 2024; Shori, 2017). Camel kefir is obtained by spontaneous fermentation of kefir grains under approximately room temperature, resulting in a thick, creamy product that is usually chilled for consumption (Hamed et al., 2024; Shori, 2017; Seifu, 2023). Other traditional fermented CM products include Laban, Shubat, Susuac, and Zrig (Muthukumaran et al., 2023; Seifu, 2023; Chammas et al., 2006).

**Table 2:** Different traditionally fermented camel milk products consumed worldwide.

Product name	Bacteria used	Fermentation & storage conditions	Additives	Texture	Reference
Chal (Bulgaria, Iran, and Turkey)	*L. plantarum, L. paraplantarum, Lb. kefiri, Lb. gasseri, Lacticaseibacillus. paracasei, Leuconostoc lactis, Weissella cibaria,* & *Enterococcus (E.) faecium*	pH 4.3–4.6, Temp 22–28 °C, incubated in earthenware jug, Stored in skin bags	None	Slightly thick	Hamed et al. (2024), Seifu (2023), and Soleymanzadeh et al. (2016)
Doogh (Iran, Afghanistan, Turkey)	*Bifidobacterium bifidum*	pH 3.64–4.2, Temp 37–38 °C, Stored at 5–7 °C,	Salt, ginger extract, pectin, gum arabic	Thin, drinkable	Azarikia and Abbasi (2010)
Gariss (Sudan)	Spontaneous fermentation	pH 3.4–3.7, Temp 30–37 °C, Ambient	None	Liquid, slightly thick	Hamed et al. (2024) and Shori (2017)
Kefir (Modified Camel Kefir)	Spontaneous fermentation	pH 4.3–4.4, Temp 20–26 °C, Refrigerated, short-term	Kefir grains	Thick, slightly creamy	Hamed et al. (2024), Shori (2017), and Seifu (2023)
Laban (Middle East, North Africa)	*S. thermophilus, Lb. bulgaricus*	pH 3.98–4.52, Temp 40–45 °C, Refrigerated	None or salt	Semi-liquid, smooth	Chammas et al. (2006)
Shubat (Kazakhstan)	Spontaneous fermentation	pH 3.7–4.1, Temp 25–30 °C, Refrigerated or ambient	None	Liquid, slightly viscous	Muthukumaran et al. (2023) and Hamed et al. (2024)
Suusac (Somalia, Kenya)	Spontaneous fermentation	pH 3.6–4.9, Temp 26–29 °C, Stored in smoked containers	Smoke-treated gourds	Thin, slightly foamy	Hamed et al. (2024) and Shori (2017)
Zrig (Mauritania)	Spontaneous fermentation	pH 4.2–4.5, Temp 25–30 °C, Consumed fresh	Sugar millet	Thin, drinkable	Seifu (2023)

**Figure 6:**
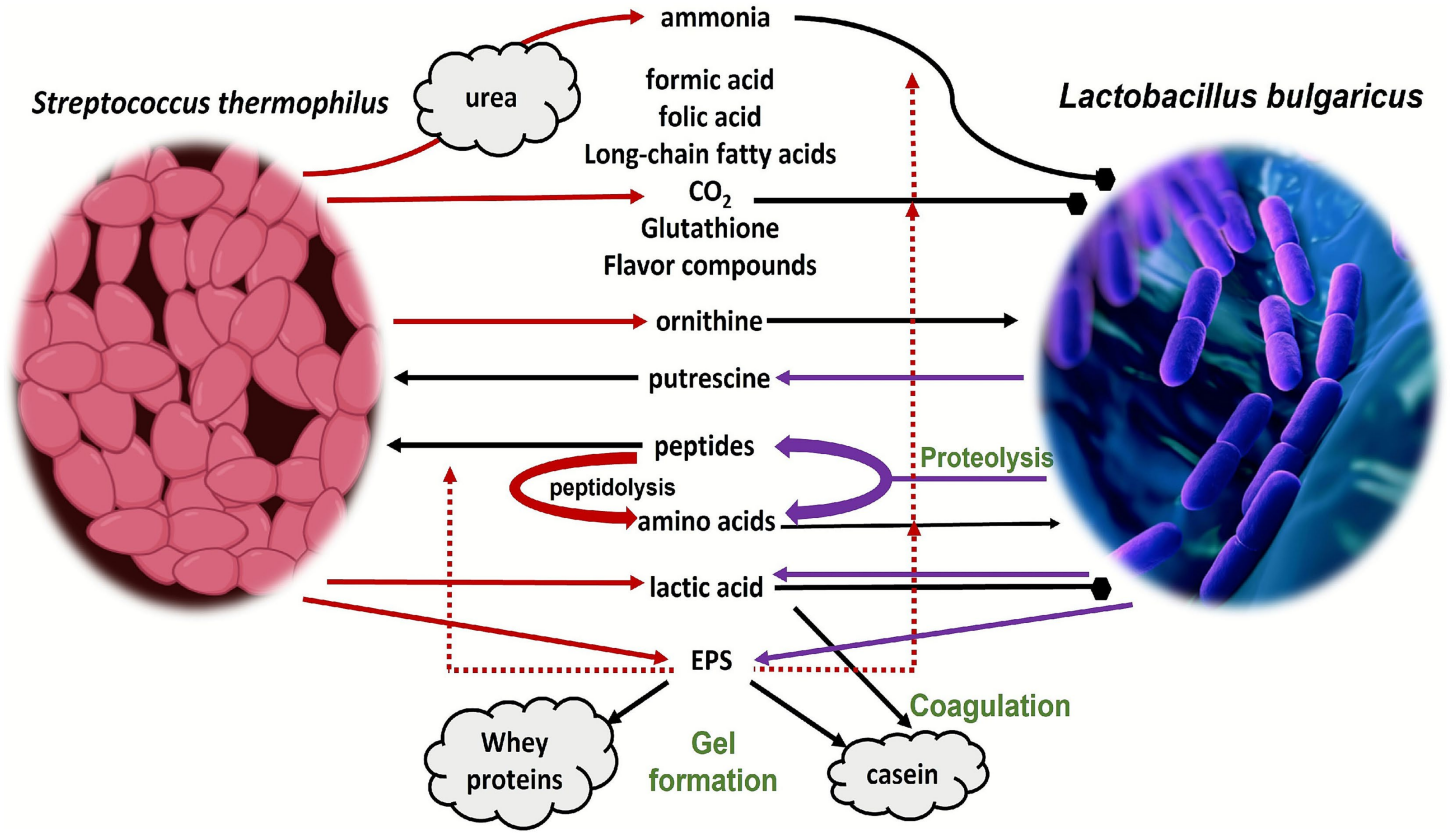
Schematic representation of the interactions between *S. thermophilus* and *Lb. bulgaricus* during yogurt fermentation and their effects on product attributes. The dotted lines are related to EPS, which are hypothesized to mediate the exchange of metabolites between the two species in proximity: production or enzymatic activity; positive effect of the component; negative effect; neutral or yet to be proven effect. LCFA: long-chain fatty acid; EPS: exopolysaccharides [adapted from Yang et al. (2025) and Ayivi and Ibrahim (2022)] under creative commons permission.

Figure 6 illustrates the mutualistic interaction between *Lb. delbrueckii* subsp. *bulgaricus* and *S. thermophilus* in the fermentation of milk. These interactions are crucial for establishing the appropriate microbial ecosystem in milk fermentation, ensuring the quality and safety of the final product (Yang et al., 2025). Synergistic interactions between these bacteria involve metabolite exchange, protein and amino acid metabolism, lactic acid and acidity regulation, urea activity, pH regulation, exopolysaccharide production, and glutathione production (Ayivi and Ibrahim, 2022). *S. thermophilus* enhances the growth of *Lb. bulgaricus* and metabolism by producing ammonia, formic acid, and folic acid, while *Lb. bulgaricus* hydrolyzes milk proteins into the growth-promoting peptides and amino acids used by *S. thermophilus* (Yang et al., 2025). *S. thermophilus* consumes oxygen (O_2_) in the fermentation medium, producing carbon dioxide (CO_2_), which is preferred by *Lb. bulgaricus* (Sasaki et al., 2014). Additionally, *Lb. bulgaricus* produces lactic acid, which adds to the acidity of the yogurt, by breaking down casein into peptides and amino acids through the expression of proteases (Ayivi and Ibrahim, 2022). *Lb. bulgaricus* and *S. thermophilus* produce long-chain fatty acids and glutathione, which maintain membrane stability and oxidative stress resistance (Yang et al., 2025). This proto-cooperation ensures optimal growth, metabolite exchange, and stability in yogurt fermentation (Ayivi and Ibrahim, 2022). Bacteria produce several fermentation products such as lactic acid, flavor components, and EPS, enhancing the yogurt’s texture and mouthfeel. Their mutual metabolic exchange ensures stability and optimal growth, driving efficient milk fermentation (Wang J. et al., 2025). This symbiosis highlights the importance of microbial interactions in achieving stable coexistence and efficient substrate conversion in food fermentation. Therefore, understanding the connection between *Lb. bulgaricus* and *S. thermophilus* may contribute to enhancing the texture, flavor, and quality of fermented dairy products by optimizing strain selection or process conditions.

**Figure 7:**
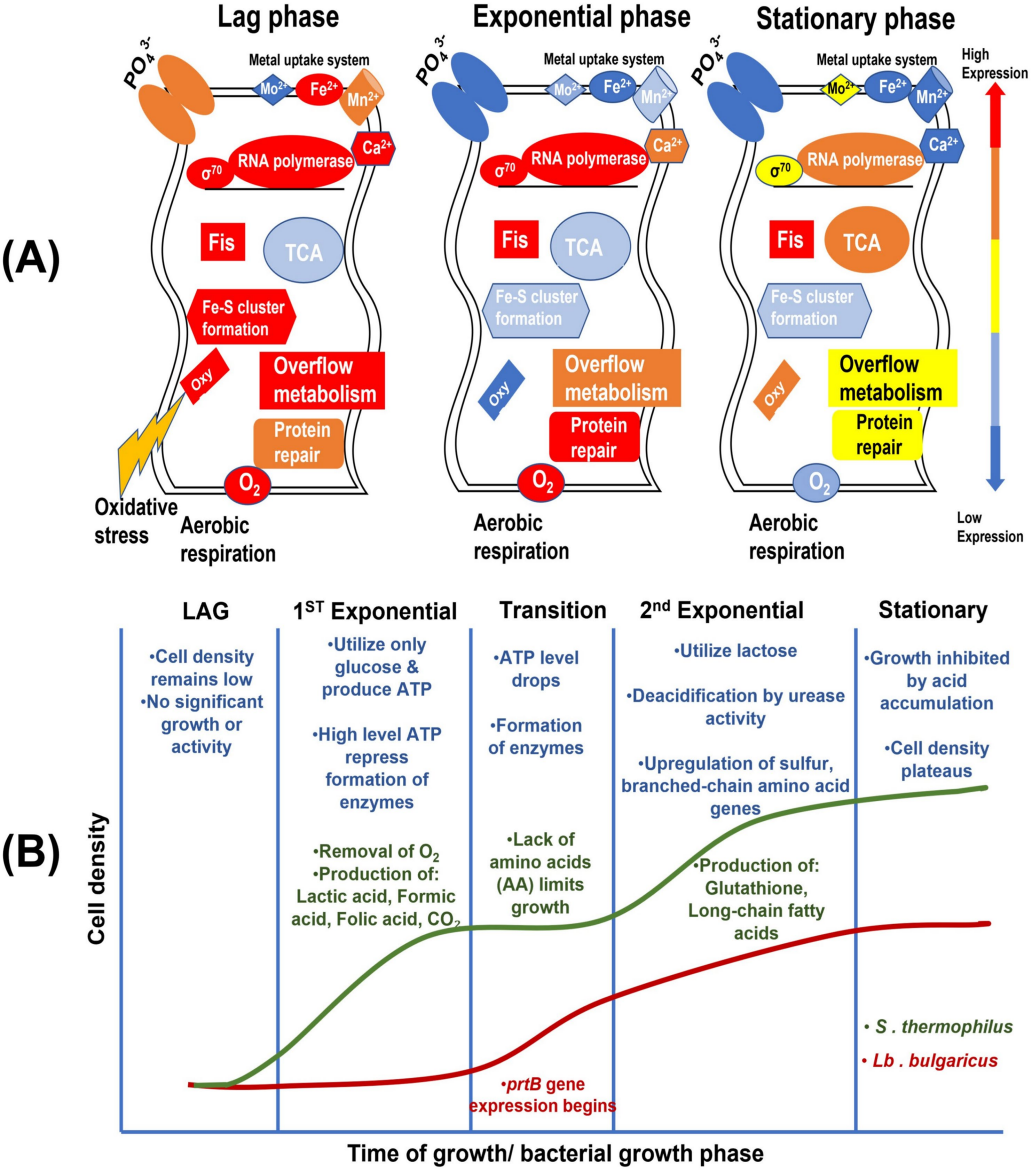
**(A)** The major physiological processes occurring during the three phases of bacterial growth (stationary, lag, and exponential). The processes and functional protein groups are depicted according to the varying levels of gene expression in each growth phase: blue, low expression; yellow, medium expression; red, high expression [adapted from Rolfe et al. (2012)] with permission from American Society for Microbiology. **(B)** Schematic diagram showing the different bacterial growth phases during milk fermentation. The growth trajectories of *S. thermophilus* and *Lb. bulgaricus* are shown by the green and red curves, respectively. AA: amino acid; EPS: exopolysaccharide; LCFA: long-chain fatty acid [adapted from Yang et al. (2025)] under creative commons permission.

As schematically outlined in Figure 7, bacterial cells quickly sense their surroundings and initiate transcription during the different phases of bacterial growth. A transcriptomic analysis of the lag phase helped to identify the regulatory mechanisms that govern the transitions from the stationary to lag phases and from the lag to exponential phases, where aerobic respiration is fully established and tricarboxylic acid (TCA) cycle enzymes and metal uptake systems support growth (Rolfe et al., 2012). The stationary phase is characterized by low TCA cycle components, active Fe–S cluster formation, strong expression of genes involved in the stress response systems and protein repair pathways, and anaerobic respiration. Increased protein repair and the production of Fe–S clusters occur as a result of the oxidative stress response that is initiated during the lag phase (Rolfe et al., 2012). Two exponential growth phases, each distinguished by significant interactions and metabolic alterations, are separated by a transition phase.

*S. thermophilus* can withstand neutral pH and efficiently uptake amino acids (Sieuwerts, 2016), which may promote its faster growth in the lag phase as an adaption to the milk environment. The first exponential phase (Figure 7) is the most common, which generates carbon dioxide, lactic acid, formic acid, and folic acid. *S. thermophilus* provides folic acid and formic acid, which *Lb. bulgaricus* requires for its growth (Lecomte et al., 2016), while the bacterium’s consumption of oxygen, and consequent production of carbon dioxide, promotes the anaerobic growth of *Lb. bulgaricus* (Sasaki et al., 2014). *S. thermophilus* hydrolyzes lactose by utilizing *β*-galactosidase, resulting in the production of glucose and galactose, which are then glycolyzed to generate lactic acid in milk (Tarrah et al., 2018). The limited availability of amino acids slows bacterial growth during the transition phase. *Lb. bulgaricus* activates the *prtB* gene, generating an extracellular protease that breaks down casein into peptides and amino acids, providing nitrogen sources to promote the growth of both bacteria (Liu et al., 2016). The second exponential phase during milk fermentation involves the growth of *Lb. bulgaricus* and *S. thermophilus*, which upregulate pathways for the synthesis of long-chain fatty acid and glutathione (Yang et al., 2025). These conditions offer advantages to the growth of *Lb. bulgaricus* by raising pH and improving acidification, lactose utilization, and lactic acid production (Yu et al., 2020). During the stationary phase, bacterial growth is delayed due to acid accumulation; nevertheless, the metabolic synergy between the two species ensures efficient fermentation (Yang et al., 2025). Future research could explore the effect of various probiotic strains on fermentation and their contributions to improving the techno-functional properties of fermented dairy products. *S. thermophilus* plays a major role in lactose utilization, producing lactic acid, which lowers pH and enhances proteolysis, resulting in the liberation of peptides and amino acids with antimicrobial and antioxidant properties (Elhamid and Elbayoumi, 2017). *S. thermophilus* also generates EPS by facilitating water-binding capacity and casein network rigidity, resulting in viscosity improvement and reducing syneresis in BM yogurt (Yang et al., 2014; Daba et al., 2021). Moreover, strains ST-3021 and ST-4845 are EPS-producing strains that enhance the hardness and texture of yogurt, resulting in a creamier yogurt with a superior mouthfeel (Folkenberg et al., 2006). The bacterium interacts synergistically with *Lb. bulgaricus,* further highlighting its importance in commercial yogurt production. Thermophilic lactobacilli, particularly *Lb. bulgaricus*, surpass the fermentation performance other bacteria by exhibiting strong proteolytic activities (proteinases and peptidases), which contribute to flavor development and the formation of bioactive peptides with antimicrobial and antioxidant properties (Kieliszek et al., 2021). Strain GL03-1 of *Lb. bulgaricus* was reported to substantially improve the viscoelastic properties of buffalo yogurt, exhibiting a three-dimensional mesh-like gel structure (Yang et al., 2014). The combination of *Lb. fermentum* and *Lb. bulgaricus* in BM yogurt increased acid generation and adhesiveness, while decreasing the degree of whey separation, offering an effective fat replacer (Tiwari et al., 2021).

Some studies have investigated the influence of other lactobacilli on the fermentation time and yogurt quality. For instance, the addition of *Lb. helveticus* H9 as a starter culture in BM yogurt fermentation led to shorter fermentation times and higher levels of volatile compounds (Zhou et al., 2019). *Lb. helveticus* is a probiotic bacterium that can be used as a starter culture in fermented CM and exhibits high proteolytic activity, producing bioactive peptides with therapeutic benefits and antibacterial properties (Chelladhurai et al., 2024). Notably, selective strains of *Lb. helveticus* are also considered for imparting nutty and anti-bitter flavors and improving the sensory appeal of fermented CM (Chelladhurai et al., 2023). *Lb. helveticus*-fermented CM exhibited high levels of auto-aggregation, coaggregation, and adhesion, demonstrating the ability to adhere to intestinal epithelial cells and offer defense against infections (Mahmoudi et al., 2019).

Probiotic *Lactobacillus* strains (*L. plantarum*-KX881772, *L. plantarum*-KX881779, *Lb. reuteri*-KX881777) of CM performed better than non-CM strains (Hati, 2018). Research on the incorporation of *L. plantarum* in CM fermentation suggests its potential for industrial utilization. *L. plantarum* HUM19, *Lb. fermentum*, and *Lb. rhamnosus* showed high proteolysis and sensory characteristics in both fermented CM and BM (Moslehishad et al., 2013). As a probiotic adjunct culture in yogurt production, *L. plantarum* exhibits strong capacity to enhance functional attributes such as flavor and probiotic viability without compromising sensory profiles, making it a suitable and considerable option for the creation of innovative functional dairy products (Li et al., 2017). *L. plantarum* exhibited proficiency in suppressing the proliferation of foodborne pathogens in fermented CM (Li et al., 2017), rendering it a secure choice as a primary fermentative. *L. plantarum* was also found to enhance secondary proteolysis in CM, releasing peptides and amino acids that act as flavor precursors (Mishra et al., 2019). Mixed starters of *L. plantarum* and *S. thermophilus* exhibited enhanced casein breakdown and modified proteolysis patterns (Li et al., 2019). Moreover, *L. plantarum* KX041 produces EPS with free radical-scavenging activity during fermentation (Xu et al., 2019). These results highlight the significance of adopting a careful approach when selecting probiotic strains for co-cultures in fermented dairy production (Li et al., 2019).

According to Bandiera et al. (2013), *Lb. casei* can be effectively incorporated into yogurt without negatively affecting other starter cultures. The probiotic strain *Lb. casei* (Bai et al., 2020) shortened the fermentation time and increased beneficial EPS production, resulting in higher bacterial viability, improved viscosity and enhanced gel structures in fermented CM (Bai et al., 2020; Lee and Lucey, 2004; Wang D. et al., 2025). *Lb. casei* and *Lb. acidophilus* enhanced the antioxidant capacity in probiotic BM yogurt by releasing potent peptides with free radical-scavenging and metal-chelating properties (Fardet and Rock, 2018; Tadesse and Emire, 2020). In commercial products such as Yakult^®^ and Actimel^™^, fermentation with *Lb. casei* alone generated volatile compounds dominated by acetic acid, acetoin, and butyric acid. When combined with other yogurt cultures, *Lb. casei* increased the levels of 3-hydroxy-2-butanone and hexanoic acid in BM yogurt, thereby enhancing the yogurt-like aroma (Chen et al., 2017; Zaręba et al., 2014). Table 3 summarizes the techno-functional effects of various LAB strains and species on the properties of yogurts and fermented camel milk products.

**Table 3:** Techno-functional properties of different milk products fermented with different types of lactic acid bacteria.

Culture	Techno-functional effects	References
*Lb. helveticus* H9	Shortens fermentation time, increases volatile components	Zhou et al. (2019)
*Lactiplantibacillus plantarum* IMAU80106, IMAU10216, IMAU70095	Increases the coagulation ability and proteolytic activity	Li et al. (2017)
*Lb. casei Zhang*	Shortens fermentation time, increases EPS production & improves texture	Bai et al. (2020)
*S. thermophilus* S-3	Decreases syneresis	Xu et al. (2018)
*S. thermophilus* CC30	Has emulsification properties	Kanamarlapudi and Muddada (2017)
*L. plantarum* C70	Improves texture & rheological properties	Ayyash et al. (2019)
*S. thermophilus* LY03	Increases apparent viscosity	De Vuyst et al. (2003)
*Lb. delbrueckii ssp. bulgaricus* DGCC291	Increases viscosity & decreases syneresis	Gentès et al. (2015)
*L. plantarum* YW11	Improves viscosity of skim milk yogurt	Wang et al. (2015)
*S. thermophilus* CH101, NIZO 2104	Increases viscosity & decreases syneresis	Gentès et al. (2015)
*Lb. casei Zhang*	Improves viscosity and promotes gel formation	Wang D. et al. (2025)
*S. thermophilus* CH101	Produces exopolysaccharides (EPS)	De Vuyst et al. (2003)
*Lb. casei LcS*	Contributes anti-obesity effects	Karimi et al. (2015)
*L. plantarum* OLL2712	Contributes anti-diabetic effects	Toshimitsu et al. (2016)
*Lb. helveticus* 881315	Produces angiotensin-converting enzyme (ACE) inhibitory peptides	Shi et al. (2016)

### 3.3 Effects of processing and fermentation conditions

In addition to the milk type and bacterial strain, the processing conditions such as heat treatment, pressure, homogenization, and additives are key variables that affect yogurt quality. Conventional heat treatments prior to fermentation have detrimental effects on the organoleptic qualities of fermented milk products (i.e., texture, creation of off-flavors, and color) (Asaduzzaman et al., 2021; Yirda et al., 2020). In addition to deactivating enzymes and extending the shelf life of milk by destroying pathogenic microbes and spoiling either completely or partially (Hattem et al., 2011), heat treatment has major effects on the composition of fermented CM and the release of bioactive peptides (Izadi et al., 2019). As previously mentioned, the denaturation of milk whey proteins and their bonding with *κ*-casein are the most significant consequences of heating milk (Genene et al., 2018). κ-Casein interacts with sulfhydryl-disulfide bonds during heating above 70 °C to produce micelle-bound and soluble thermal co-aggregates (Asaduzzaman et al., 2021), which has an impact on the protein network and the gelation characteristics of casein micelles (Mohamed et al., 2022b). Compared to BM, CM was reported to be less stable and more susceptible to heat treatments (Mohamed et al., 2022b). A typical pasteurization procedure for CM is 60 °C for 30 min, 63 °C for 30 min, and 75 °C for 15 s (Alhaj et al., 2013). Thermally treated CM (63 °C for 30 min, 72 °C for 15 s, and 100.5 °C for 10 min) reduced the overall acceptability, taste score, and texture compared to those of untreated milk (Lund et al., 2019). Moreover, CM exhibited poor heat stability when autoclaved for 15 min at 121 °C, resulting in sedimentation and whey separation (Alhaj et al., 2013). Prior heating of the milk for 10–15 min at 80 °C–95 °C promotes protein network development, water retaining capabilities, enzyme denaturation, and the destruction of unavoidable microbes. Whey separation and textural flaws may be reduced by optimizing the incubation temperature (∼40 °C), which can eventually improve the yogurt texture (Lee and Lucey, 2004).

Table 4 shows the effects of various fermentation pre-treatments, including thermal processing, homogenization, HPP, fermentation, and thermosonication (Kenari and Razavi, 2021). By adopting non-thermal technology, most of the adverse effects of thermal treatment can be avoided, leading to higher-quality food products (Ahmad et al., 2019). Milk homogenization was reported to improve BM yogurt quality by reducing the size of fat globules (Ho et al., 2022b; Trujillo et al., 2016). However, since CM is naturally characterized by smaller and more homogeneous fat globules than BM, it does not require homogenization (Table 1). HPP can enhance the rheological characteristics of fermented BM by modifying milk proteins, such as whey protein denaturation and casein micelle disruption due to colloidal calcium phosphate solubilization (Nassar et al., 2020). HPP treatment at 300 and 600 mPa for 10 min at 10 °C could maintain the greatest bacterial counts. While high-temperature short-time and UHT treatments increase the particle size in BM, the opposite effect is found in CM (Ayyash et al., 2022). HPP treatment lowered the particle size in both CM and BM and produced yogurts with creamier mouthfeels and smoother textures (Ayyash et al., 2022). Furthermore, fermented heat-treated CM produced stronger gels with better storage properties and less modulus loss than HPP-treated milk, notably with treatment at 85 °C, whereas ultrafiltration increased only the viscosity and not the gel strength (Sobti et al., 2024). Therefore, HPP is inferior to heat treatment in enhancing fermented CM texture. Thus, the observed difference can be attributed to the differences between CM and BM in the original micelle structure and how these are affected by heating and HPP treatments.

**Table 4:** The effect of pre-fermentation conditions on the quality of fermented camel milk (CM) products.

Treatment	Key findings	References
Prior heating (85 °C, 30 min)	Heat-treatment enhanced the texture and rheological properties signifying stronger gels.	Sobti et al. (2024)
Ultra-high temperature (UHT-140 °C, 3 s)	UHT & HPP increased the viscosity and HPP improved the rheological properties (G′ and G′′)	Ayyash et al. (2022)
High-Pressure Processing (HPP) (350/550 MPa, 5 min)	HPP-treated fermented CM had lower viscosity than heat-treated milk	Ayyash et al. (2022) and Sobti et al. (2024)
Fermentation temperature (40 °C, pH 4.6)	Lower incubation temperatures increased storage modulus & yield stress and reduced whey separation & textural defects	Lee and Lucey (2004)
Thermosonication (75% amplitude, 55 °C, 10 min)	Reduced syneresis and improved acidity, texture, flavor, & color	Kenari and Razavi (2021)

The combination of low incubation temperatures and moderate to high inoculation rates can enhance the gel strength and storage modulus, thereby enhancing the texture of BM yogurt (Lee and Lucey, 2004). In the dairy sector, the application of ultrasound has been used to regulate the functional qualities of dietary proteins as well as the microstructure and texture of fat-containing items, including yogurt, cheese, and ice cream (Ahmad et al., 2019). Moreover, ultrasound inactivates microorganisms and enzymes (Ahmad et al., 2019), develops a sweetening effect in yogurt (Wu et al., 2000), enhances emulsification and homogenization by decreasing the size of milk fat globules, and shortens fermentation times by enhancing lactose hydrolysis in yogurt production (Akdeniz and Akalın, 2019). High-intensity ultrasound treatment was reported to improve various characteristics of BM, including the denaturation of casein micelles, formation of aggregates between *κ*-casein and whey proteins (*β*-lactoglobulin), viscosity, texture, fat globule size, and surface membrane area (Akdeniz and Akalın, 2019).

**Figure 8:**
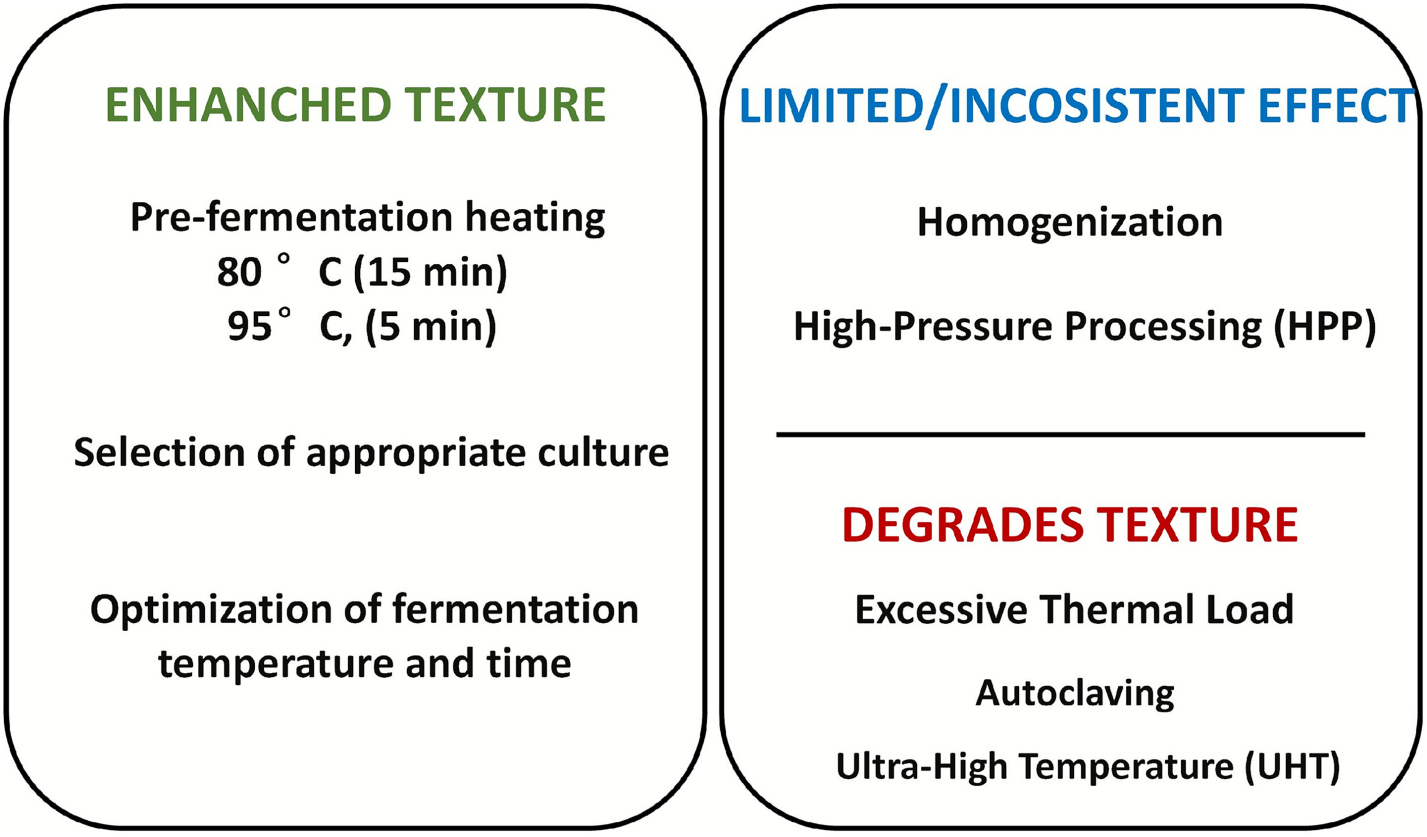
A schematic overview of processing and fermentation condition that enhance, have limited influence on, and degrade the texture of fermented camel milk.

Thermosonication considerably improved the qualities of fermented CM, providing a potentially viable method for improving yogurt quality. The ideal parameters for thermosonication of CM are 55 °C for 10 min at 75 amplitudes (Kenari and Razavi, 2021). The texture of set and stirred yogurt was also influenced by sonication during BM fermentation, which may result in improved smoothness and creaminess (Körzendörfer et al., 2017). By comprehensively understanding the impacts of manufacturing processes on the final product, the processes may be optimized to produce fermented CM with desired textural features. Figure 8 presents a summary overview of processing and fermentation conditions that enhance, have limited influence on, degrades the quality of fermented CM.

### 3.4 Effect of additives, coagulants, and texture enhancers

Table 5 summarizes the effects of coagulants and texture-enhancing additives on the quality of fermented products: these additives result in more calcium, phosphorus, and saturated fatty acids in fermented CM, whereas the protein, lactose, and total solids are enhanced in BM yogurt, contributing to its preferred flavor and texture (Galeboe et al., 2018). Fortifying CM with milk from other species such as sheep, buffalo, and bovine has been proposed as a potential strategy to enhance the texture and minimize syneresis (Kamal-Eldin et al., 2020; Ait El Alia et al., 2023; Ibrahem and El Zubeir, 2016). Compared to the yogurt produced entirely with CM, yogurt manufactured using a combination of CM and BM had better physicochemical properties and sensory profile (Kamal-Eldin et al., 2020; Mustafa, 2015). Sheep milk (40–60%) (Ibrahem and El Zubeir, 2016) and buffalo milk (90%) (Khalifa and Zakaria, 2018) increased the total solids content and acceptability of fermented CM, while oat milk (40%) improved the antioxidants, viscosity, and sensory qualities (Atwaa et al., 2020). The enhancement of fermented CM quality by the fortification with milks from other animals was attributed to the differences in composition of third portions, especially the contents of *β*-lactoglobulin and *κ*-casein. Fortification of CM with BM powder improved the hardness and consistency of fermented CM gels and minimize syneresis during the processing and storage phases (Sobti and Kamal-Eldin, 2019; Atwaa et al., 2020; Salih and Hamid, 2013; Omar et al., 2019). Acid gelation of fermented CM was shown be aided by sodium caseinate prepared from CM casein micelles, which lead to improved texture and rheological properties of the gel (Wang J. et al., 2025), suggesting that casein source and structural compatibility are significant factors for effective coagulation. Supplementation with high levels of casein or whey protein was shown to increase syneresis by creating larger porous and less linked aggregates (Puvanenthiran et al., 2002) suggesting the need for optimized levels of added milk proteins.

**Table 5:** The effect of texture-enhancing additives on fermented camel milk (CM).

Additive	Rheology	Syneresis	Sensory acceptability	Mechanism	References
Bovine milk & powder	Gel strength, firmness & viscosity improved	Reduced	Improved	Provides β-lactoglobulin and κ-casein enhancing protein network, increased total solids	Kamal-Eldin et al. (2020) and Mustafa (2015)
Buffalo milk (90%)	Firmness & viscosity improved	Reduced	Improved	High protein and fat improve network density	Khalifa and Zakaria (2018)
Sheep milk (40–60%)	Firmness & viscosity improved	Reduced	Improved	Increased total solids, proteins & fat strengthen gel	Ibrahem and El Zubeir (2016)
Oat milk (40%)	Viscosity improved	Reduced	Improved	Increased phenolic content, antioxidants, and dietary fiber contents	Atwaa et al. (2020)
BM casein (2.5%) & whey protein (4%)	Gel strength, firmness & viscosity improved	Reduced up to optimal level	Reduced up to optimal level	Additional milk proteins enhance protein–protein interactions and network continuity	Sobti and Kamal-Eldin (2019)
Sodium caseinate (2.5%)	Firmness and water holding capacity increased	Reduced	Improved	Caseinate improves acid-induced gelation by reinforcing casein network	Wang J. et al. (2025)
Pea protein (3–6%)	Gelation and viscosity improved	Reduced	Improved	Plant proteins reinforce protein–protein interactions	Olaimat et al. (2023)
Gelatin (0.75–1.0%)	Hardness and viscosity improved	Reduced	-	Thermoreversible gel traps water in matrix	Ho et al. (2022a) and Mudgil et al. (2018)
Sodium alginate (0.3 and 0.5%)	Viscosity improved	Reduced	Improved	Ionic gelation and water entrapment, interaction with protein network	Sobti et al. (2021)
Sodium alginate (0.75%) and CaCl₂ (0.075%)	Firmness improved	Increased	Improved	Increased ionic calcium from CaCl₂ promoted excessive protein aggregation, resulting in coarse gels and increased syneresis	Hashim et al. (2009)
Gum arabic (1–2%)	Texture and viscosity improved	Reduced	Improved	Hydrocolloid increases water-binding and stabilizes protein matrix	Jasim et al. (2018)
Xanthan gum (0.75%)	Texture, viscosity improved	Reduced	Improved	Thickening via polysaccharide-water interactions	Mohsin et al. (2019)
Oat β-glucan (2%)	Viscosity, water holding capacity improved	Reduced	Improved	β-glucan enhances water-holding capacity and serum viscosity	Ladjevardi et al. (2016)
Modified starch (3%)	Viscosity increased	Reduced	Improved	Starch granules bind free water and reinforce gel structure	Ibrahim and Khalifa (2015)
Modified starch (2.5%) and CaCl2 (0.075%)	Viscosity improved	Reduced	-	CaCl2 increased viscosity but also syneresis, stabilized by starch. Modified starch > corn starch.	Oselu et al. (2022b)
Sweet Potato Powder (3%)	Viscosity slightly increased	Reduced	Improved	Dietary fibers enhance water retention and gel stability	Omar et al. (2019)
Monk fruit sweetener (0.42–2.54%)	Viscosity improved	-	Altered color and sweetness	Sweetener modifies serum phase and total solids	Buchilina and Aryana (2021)
Carob fiber (2%),	Cohesiveness improved	Reduced	Improved	Dietary fiber strengthens matrix and improves water binding	Jrad et al. (2021)
Date and orange fibers (4.5%)	Texture, viscosity increased	Reduced	Orange fiber improved flavor and appearance	Orange fibers increase total solids and stabilize gel network than date fibers	Ibrahim and Khalifa (2015)
Persimmon pulp (5%)	Gel strength improved	Reduced	Improved	Pulp polysaccharides promote gel formation and shorten fermentation time	Alia et al. (2023)
Whey protein isolate (WPI-3%) + κ-carrageenan (0.1%) + traditional samphire molasses (TSM-3%) + xanthan gum (0.5%)	Significantly viscosity increased	Reduced	Improved	Synergistic protein–polysaccharide interactions strengthen gel matrix	Kavas (2016)
Microbial Transglutaminase (MTGase, 0.4%)	Viscosity and gel density improved	Reduced	-	Covalent cross-linking of milk proteins strengthens gel network	Abou-Soliman et al. (2017)
MTGase & WPC (6.2%)	Gel structure improved	Reduced	Improved	Combined cross-linking and protein enrichment enhance matrix strength	Bulca et al. (2022)
Transglutaminase enzyme, glutathione, & gelatin (0.5–1.0 g/300 mL)	Moderate increase in firmness	Reduced	Improved slightly	Enzymatic cross-linking with auxiliary stabilizers improves water retention	Arslan Amin et al. (2023)
Trisodium citrate (30 mmol/L) + MTGase	Texture improved	Reduced	Neutral	Citrate dissociates micelles, improving MTGase accessibility	Chen et al. (2019)
Fruit purees (15, 25, 25% apricot, blueberry, mango, peach, pineapple, and strawberry)	Gel structures, viscosity improved	Reduced	Improved	Fruit solids and fibers enhance gel structure and viscosity	Sobti et al. (2023)

Efforts have been made to improve fermented CM quality by adding hydrocolloids such as gelatin, sodium alginate, gum Arabic, and xanthan gum (Table 5). Gelatin (0.75–1.0%) (Mudgil et al., 2018), and xanthan gum (0.75%) (Mohsin et al., 2019), oat β-glucan (2%) (Ladjevardi et al., 2016), modified starch (3%) (Ibrahim and Khalifa, 2015) improved hardness, viscosity, water holding capacity, and sensory perception and sweet potato powder (3%) reduced syneresis and extended shelf-life (Omar et al., 2019). Adding hydrocolloids (gelatin, alginate, gum Arabic, and pectin) to CM containing casein, whey protein, and calcium chloride produced different effects. While alginate and pectin improved texture and rheology and reduced syneresis, gum Arabic and gelatin had small negative effect on hardness and rheology (Sobti et al., 2020). When mixed with hydrocolloids (gelatin, gum Arabic, pectin, and sodium alginate), calcium chloride (CaCl₂) was shown to cause excessive protein aggregation in fermented CM leading to coarse gels, enhanced syneresis, and unwanted stiffness, which reduced consumer acceptance (Sobti et al., 2020; Oselu et al., 2022b; Ramasubramanian et al., 2008). Fat substitutes such as gelatin, k-carrageenan, pectin, inulin, fibers, and starch increased the viscosity, decreased syneresis, and improved overall sensory qualities (Saleh et al., 2018; Atwaa et al., 2020). Some ingredients were effective primarily in combination, such as whey protein isolate (3%) with *κ*-carrageenan (0.1%), samphire molasses (3%), and xanthan gum (0.5%) improved rheological properties (Kavas, 2016).

Enzymatic modifications are also being tested to improve the quality and properties of fermented CM products. For example, microbial transglutaminase (MTGase, EC 2.3.2.13), a natural alternative to gelatin for yogurt stabilization, proved to be more effective than the dairy powder components (Abou-Soliman et al., 2017). MTGase was shown to enhance the structural, textural, and sensory qualities of fermented CM by improving the microstructure and volatile compounds content (Bulca et al., 2022; Chen et al., 2019; Hovjecki et al., 2021). MTGase is commonly added to fermented dairy products at below 1% concentration (Arslan Amin et al., 2023) to strengthen the gel matrix by generating cross links between lysine and glutamate residues of proteins (Gharibzahedi and Chronakis, 2018). However, MTGase alone cannot fully improve the poor texture of fermented CM, necessitating milk protein enrichment (Abou-Soliman et al., 2017; Ma et al., 2019; Metwalli et al., 2023; Han et al., 2020). Further research is needed to optimize the texture of fermented MTGase-treated CM and testing its interaction with milk proteins and hydrocolloids.

Fruit-based ingredients remain popular among consumers because of added color, flavor, taste, and texture (Alia et al., 2023; Sobti et al., 2023; Ramasubramanian et al., 2008; Kavas, 2016). Flavored CM labans supplemented with fruit purees (25% apricot, blueberry, mango, peach, pineapple, and strawberry) showed higher storage moduli, loss moduli, and viscosity values; enhanced gel structures; and improved acceptable sensory properties (Sobti et al., 2023). Monk fruit sweetener (0.42–2.54%) can be used in fermented CM to change its color and viscosity, while providing a calorie-free, healthy alternative to traditional sweeteners (Buchilina and Aryana, 2021). Plant-based additives including pea protein (3–6%) resulted in enhancement of yogurt texture and antioxidant qualities (Olaimat et al., 2023), while carob fiber (2%) improved cohesion and water-holding capacity (Jrad et al., 2021). Date and orange dietary fiber (4.5%) also improved the texture and probiotic development (Ibrahim and Khalifa, 2015) and persimmon pulp (5%) promoted gel formation (Alia et al., 2023).

## 4 Conclusion

Fermented camel milk has been shown to have several health benefits including anti-diabetic, anti-hypercholesterolemic, anti-oxidative activity. Unlike BM, the fermentation of CM does not afford a set yogurt but rather a liquid-like that is better described as “drinkable yogurt.” Although it is accepted that the absence of *β*-lactoglobulin and the low level of *κ*-casein are major contributors to the weak gels in fermented CM products, other components of CM such as the high contents of β-casein and proteolytic products may contribute to this difference. The mechanisms of the interactions between these biochemical constituents and how they collectively limit protein interactions, micellar crosslinking, and network formation during acid gelation are not yet understood. The contribution of mineral equilibria, casein phosphorylation, β-casein hydrophobicity, and proteolysis kinetics in the production of CM gels still remain to be explored. Efforts are still needed for the optimization of protein composition, pre-fermentation heat treatment, selection of appropriate bacterial cultures and fermentation conditions, and additives in order to improve appearance, texture, and viscosity. In the future, it is more practical to concentrate product development efforts on improving premium drinkable or semi-liquid fermented CM products rather than adapting to traditional BM set yogurts. The fermented CM flavor can be enhanced by adding fruit purees, and certain herbs and spices.

## References

[ref1] Abd El-Aziz M Kassem JM Aasem FM Abbas HM. (2022). Physicochemical properties and health benefits of camel milk and its applications in dairy products: a review. Egypt. J. Chem. Available online at: https://journals.ekb.eg/article_203612.html

[ref2] Abdeldaiem A. Ali A. Shah N. Ayyash M. Mousa A. (2022). Physicochemical analysis, rheological properties, and sensory evaluation of yogurt drink supplemented with roasted barley powder. LWT 173:114319. doi: 10.1016/j.lwt.2022.114319

[ref3] Abou-Soliman N. H. I. Sakr S. S. Awad S. (2017). Physico-chemical, microstructural and rheological properties of camel-milk yogurt as enhanced by microbial transglutaminase. J. Food Sci. Technol. 54, 1616–1627. doi: 10.1007/s13197-017-2593-9, 28559621 PMC5430194

[ref4] Ahmad T. Butt M. Z. Aadil R. M. Inam-ur-Raheem M. Abdullah Bekhit A. E. D. . (2019). Impact of nonthermal processing on different milk enzymes. Int. J. Dairy Technol. 72, 481–495. doi: 10.1111/1471-0307.12622

[ref6] Ait El Alia O. Zine-Eddine Y. Ajbli N. Kzaiber F. Abdelkhalek O. Boutoial K. (2023). Optimization of camel milk coagulation: the use of coagulants of microbiological and plant origin. Acta Sci. Pol. Technol. Aliment. 22, 81–91. doi: 10.17306/J.AFS.2023.1106

[ref7] Ait El Alia O. Zine-Eddine Y. Chaji S. Boukrouh S. Boutoial K. Faye B. (2025). Global camel milk industry: a comprehensive overview of production, consumption trends, market evolution, and value chain efficiency. Small Rumin. Res. 243:107441. doi: 10.1016/j.smallrumres.2025.107441

[ref8] Akdeniz V. Akalın A. (2019). New approach for yoghurt and ice cream production: high-intensity ultrasound. Trends Food Sci. Technol. 86, 392–398. doi: 10.1016/j.tifs.2019.02.046

[ref9] Alhaj O. A. Taufik E. Handa Y. Fukuda K. Saito T. Urashima T. (2013). Chemical characterisation of oligosaccharides in commercially pasteurised dromedary camel (*Camelus dromedarius*) milk. Int. Dairy J. 28, 70–75. doi: 10.1016/j.idairyj.2012.08.008

[ref10] Alia O. A. E. Zine-Eddine Y. Chaji S. Souhassou S. Kzaiber F. Oussama A. . (2023). Physico-chemical and sensory characterization of camel milk yogurt enriched with persimmon (*Diospyros kaki*) fruit. Acta Sci. Pol. Technol. Aliment. 22, 267–278. doi: 10.17306/J.AFS.2023.1152

[ref11] Almasri R. S. Bedir A. S. Ranneh Y. K. El-Tarabily K. A. Al Raish S. M. (2024). Benefits of camel milk over cow and goat milk for infant and adult health in fighting chronic diseases: a review. Nutrients 16:3848. doi: 10.3390/nu16223848, 39599634 PMC11597306

[ref12] Al-Zoreky N. Al-Otaibi M. (2015). Suitability of camel milk for making yogurt. Food Sci. Biotechnol. 24, 601–606. doi: 10.1007/s10068-015-0078-z

[ref13] Anema S. G. (2021). Heat-induced changes in caseins and casein micelles, including interactions with denatured whey proteins. Int. Dairy J. 122:105136. doi: 10.1016/j.idairyj.2021.105136

[ref14] Ansari F. Pourjafar H. Samakkhah S. A. Mirzakhani E. (2024). An overview of probiotic camel milk as a nutritional beverage: challenges and perspectives. Food Sci. Nutr. 12, 6123–6141. doi: 10.1002/fsn3.4298, 39554333 PMC11561798

[ref15] Arab M. Yousefi M. Khanniri E. Azari M. Ghasemzadeh-Mohammadi V. Mollakhalili-Meybodi N. (2023). A comprehensive review on yogurt syneresis: effect of processing conditions and added additives. J. Food Sci. Technol. 60, 1656–1665. doi: 10.1007/s13197-022-05403-6, 37187980 PMC10169984

[ref16] Arain M. A. Khaskheli G. B. Shah A. H. Marghazani I. B. Barham G. S. Shah Q. A. . (2023a). Nutritional significance and promising therapeutic/medicinal application of camel milk as a functional food in human and animals: a comprehensive review. Anim. Biotechnol. 34, 1988–2005. doi: 10.1080/10495398.2022.2059490 35389299

[ref17] Arain M. A. Rasheed S. Jaweria A. Khaskheli G. B. Barham G. S. Ahmed S. (2023b). A review on processing opportunities for the development of camel dairy products. Food Sci. Anim. Resour. 43, 383–401. doi: 10.5851/kosfa.2023.e13, 37181214 PMC10172818

[ref18] Arslan Amin H. M. Inayat S. Gulzar N. Bhatti J. A. Masood S. Ayub A. . (2023). Addition of transglutaminase enzyme in camel milk yoghurt to increase its sensorial aspects. Braz. J. Biol. 84:e269043. doi: 10.1590/1519-6984.269043, 36700587

[ref19] Asaduzzaman M. Mahomud M. S. Haque M. E. (2021). Heat-induced interaction of milk proteins: impact on yoghurt structure. Int. J. Food Sci. 2021:5569917. doi: 10.1155/2021/5569917 34604378 PMC8483934

[ref20] Attia H. Kherouatou N. Dhouib A. (2001). Dromedary milk lactic acid fermentation: microbiological and rheological characteristics. J. Ind. Microbiol. Biotechnol. 26, 263–270. doi: 10.1038/sj.jim.7000111, 11494100

[ref21] Atwaa E. Hassan M. Ramadan M. (2020). Production of probiotic stirred yoghurt from camel Milk and oat Milk. J. Food Dairy Sci. 11, 259–264. doi: 10.21608/jfds.2020.118366

[ref22] Ayivi R. D. Ibrahim S. A. (2022). Lactic acid bacteria: an essential probiotic and starter culture for the production of yoghurt. Int. J. Food Sci. Technol. 57, 7008–7025. doi: 10.1111/ijfs.16076

[ref23] Ayyash M. Abdalla A. Abu-Jdayil B. Huppertz T. Bhaskaracharya R. Al-Mardeai S. . (2022). Rheological properties of fermented milk from heated and high pressure-treated camel milk and bovine milk. LWT 156:113029. doi: 10.1016/j.lwt.2021.113029

[ref24] Ayyash M. Abu-Jdayil B. Itsaranuwat P. Galiwango E. Rosa C. Abdullah H. . (2019). Characterization, bioactivities, and rheological properties of exopolysaccharide produced by novel probiotic *Lactobacillus plantarum* C70 isolated from camel milk. Int. J. Biol. Macromol. 144, 938–946. doi: 10.1016/j.ijbiomac.2019.09.171 31672637

[ref25] Azarikia F. Abbasi S. (2010). On the stabilization mechanism of Doogh (Iranian yoghurt drink) by gum tragacanth. Food Hydrocoll. 24, 358–363. doi: 10.1016/j.foodhyd.2009.11.001

[ref26] Bai M. Huang T. Guo S. Wang Y. Wang J. Kwok L. Y. . (2020). Probiotic *Lactobacillus casei* Zhang improved the properties of stirred yogurt. Food Biosci. 37:100718. doi: 10.1016/j.fbio.2020.100718

[ref27] Bandiera N. S. Carneiro I. da Silva A. S. Honjoya E. R. de Santana E. H. W. Aragon-Alegro L. C. . (2013). Viability of probiotic *Lactobacillus casei* in yoghurt: defining the best processing step to its addition. Arch. Latinoam. Nutr. 63, 58–63. doi: 10.37527/2013.63.1.008, 24167959

[ref28] Benmeziane-Derradji F. (2021). Evaluation of camel milk: gross composition—a scientific overview. Trop. Anim. Health Prod. 53:308. doi: 10.1007/s11250-021-02689-0, 33961132

[ref29] Bierzuńska P. Cais-Sokolińska D. Yiğit A. (2019). Storage stability of texture and sensory properties of yogurt with the addition of polymerized whey proteins. Foods 8:548. doi: 10.3390/foods8110548, 31689896 PMC6915489

[ref30] Bintsis T. (2018). Lactic acid bacteria: their applications in foods. J. Bacteriol. Mycol. Open Access. 6, 89–94. doi: 10.15406/jbmoa.2018.06.00182

[ref31] Buchilina A. Aryana K. (2021). Physicochemical and microbiological characteristics of camel milk yogurt as influenced by monk fruit sweetener. J. Dairy Sci. 104, 1484–1493. doi: 10.3168/jds.2020-18842 33309375

[ref32] Bulca S. Umut F. Koç A. (2022). The influence of microbial transglutaminase on camel milk yogurt. LWT 160:113339. doi: 10.1016/j.lwt.2022.113339

[ref33] Chammas G. Saliba R. Corrieu G. Béal C. (2006). Characterisation of lactic acid bacteria isolated from fermented milk “laban.”. Int. J. Food Microbiol. 110, 52–61. doi: 10.1016/j.ijfoodmicro.2006.01.043, 16701913

[ref34] Chelladhurai K. Ayyash M. Turner M. Kamal-Eldin A. (2023). *Lactobacillus helveticus*: health effects, current applications, and future trends in dairy fermentation. Trends Food Sci. Technol. 136. doi: 10.1016/j.tifs.2023.04.013

[ref35] Chelladhurai K. Warakaulle S. Ali S. Turner M. Ayyash M. Kamal-Eldin A. (2024). Differences in the growth, acidification, and proteolytic activities of *Lactobacillus helveticus*, *Lactobacillus delbrueckii* subsp. *bulgaricus*, and *Streptococcus salivarius* subsp. *thermophilus* in camel and bovine milk fermentation. Int. Dairy J. 160:106075. doi: 10.1016/j.idairyj.2024.106075

[ref36] Chen C. Wang P. Zhang N. Zhang W. Ren F. (2019). Improving the textural properties of camel milk acid gel by treatment with trisodium citrate and transglutaminase. LWT 103, 53–59. doi: 10.1016/j.lwt.2018.12.063

[ref37] Chen C. Zhao S. Hao G. Yu H. Tian H. Zhao G. (2017). Role of lactic acid bacteria on the yogurt flavour: a review. Int. J. Food Prop. 20, S316–S330. doi: 10.1080/10942912.2017.1295988

[ref38] Chever S. Guyomarc’H F. Beaucher E. Famelart M. H. (2014). High-protein fat-free acid milk gels: control of protein composition and heat treatment. Int. Dairy J. 37, 95–103. doi: 10.1016/j.idairyj.2014.02.011

[ref39] Claeys W. L. Verraes C. Cardoen S. De Block J. Huyghebaert A. Raes K. . (2014). Consumption of raw or heated milk from different species: an evaluation of the nutritional and potential health benefits. Food Control 42, 188–201. doi: 10.1016/j.foodcont.2014.01.045

[ref40] Costa M. P. Rosario A. I. L. S. Silva V. L. M. Vieira C. P. Conte-Junior C. A. (2022). Rheological, physical and sensory evaluation of low-fat Cupuassu goat Milk yogurts supplemented with fat replacer. Food Sci Anim Resour. 42, 210–224. doi: 10.5851/kosfa.2021.e64, 35310563 PMC8907797

[ref41] Daba G. M. Elnahas M. O. Elkhateeb W. A. (2021). Contributions of exopolysaccharides from lactic acid bacteria as biotechnological tools in food, pharmaceutical, and medical applications. Int. J. Biol. Macromol. 173, 79–89. doi: 10.1016/j.ijbiomac.2021.01.110, 33482209

[ref42] Dalgleish D. G. Corredig M. (2012). The structure of the casein micelle of milk and its changes during processing. Annu. Rev. Food Sci. Technol. 3, 449–467. doi: 10.1146/annurev-food-022811-101214, 22385169

[ref43] de Kruif C. G. Zhulina E. B. (1996). κ-Casein as a polyelectrolyte brush on the surface of casein micelles. Colloids Surf. A Physicochem. Eng. Asp. 117, 151–159. doi: 10.1016/0927-7757(96)03696-5

[ref44] De Vuyst L. Zamfir M. Mozzi F. Adriany T. M M. Degeest B. . (2003). Exopolysaccharide-producing *Streptococcus thermophilus* strains as functional starter cultures in the production of fermented milks. Int. Dairy J. 13, 707–717. doi: 10.1016/S0958-6946(03)00105-5

[ref45] Donato L. Alexander M. Dalgleish D. G. (2007). Acid gelation in heated and unheated milks: interactions between serum protein complexes and the surfaces of casein micelles. J. Agric. Food Chem. 55, 4160–4168. doi: 10.1021/jf063242c, 17439142

[ref46] Dreiucker J. Vetter W. (2011). Fatty acids patterns in camel, moose, cow and human milk as determined with GC/MS after silver ion solid phase extraction. Food Chem. 126, 762–771. doi: 10.1016/j.foodchem.2010.11.061

[ref47] El-Agamy E. (2000). Effect of heat treatment on camel milk proteins with respect to antimicrobial factors: a comparison with cows’ and buffalo milk proteins. Food Chem. 68, 227–232. doi: 10.1016/S0308-8146(99)00199-5

[ref48] Elagamy E. I. Ruppanner R. Ismail A. Champagne C. P. Assaf R. (1996). Purification and characterization of lactoferrin, lactoperoxidase, lysozyme and immunoglobulins from camel’s milk. Int. Dairy J. 6, 129–145. doi: 10.1016/0958-6946(94)00055-7

[ref49] Elhamid A. Elbayoumi M. (2017). Effect of heat treatment and fermentation on bioactive behavior in yoghurt made from camel milk. Am. J. Food Sci. Technol. 5, 109–116. doi: 10.12691/ajfst-5-3-6

[ref50] Ereifej K. I. Alu’datt M. H. AlKhalidy H. A. Alli I. Rababah T. (2011). Comparison and characterisation of fat and protein composition for camel milk from eight Jordanian locations. Food Chem. 127, 282–289. doi: 10.1016/j.foodchem.2010.12.112

[ref51] Fabiano G. Pelham W. Majumdar A. Evans S. Manos M. Caserta D. . (2013). Elementary and middle school teacher perceptions of attention-deficit/hyperactivity disorder prevalence. Child Youth Care Forum 42. doi: 10.1007/s10566-013-9194-1

[ref52] Fang T. Guo M. (2019). Physicochemical, texture properties, and microstructure of yogurt using polymerized whey protein directly prepared from cheese whey as a thickening agent. J. Dairy Sci. 102, 7884–7894. doi: 10.3168/jds.2018-16188, 31301832

[ref53] Fardet A. Rock E. (2018). In vitro and in vivo antioxidant potential of milks, yoghurts, fermented milks and cheeses: a narrative review of evidence. Nutr. Res. Rev. 31, 52–70. doi: 10.1017/S0954422417000191, 28965518

[ref54] Folkenberg D. Dejmek P. Skriver A. Guldager H. Ipsen R. (2006). Sensory and rheological screening of exopolysaccharide producing strains of bacterial yoghurt cultures. Int. Dairy J. 16, 111–118. doi: 10.1016/j.idairyj.2004.10.013

[ref55] Galeboe O. Seifu E. Sekwati-Monang B. (2018). Production of camel milk yoghurt: physicochemical and microbiological quality and consumer acceptability. Int. J. Food Stud. 7, 51–63. doi: 10.7455/ijfs/7.2.2018.a5

[ref56] Gandhi A. Shah N. P. (2014). Cell growth and proteolytic activity of *Lactobacillus acidophilus*, *Lactobacillus helveticus*, *Lactobacillus delbrueckii* ssp. bulgaricus, and *Streptococcus thermophilus* in milk as affected by supplementation with peptide fractions. Int. J. Food Sci. Nutr. 65, 937–941. doi: 10.3109/09637486.2014.945154, 25095898

[ref57] Gazi I. Huppertz T. (2015). Casein-whey protein interactions for optimizing milk protein functionality. Agro Food Ind Hi Tech 26, 11–14.

[ref58] Genene A. Hansen E. Guya M. Hailu Y. Ipsen R. (2018). Effect of heat treatment on denaturation of whey protein and resultant rennetability of camel milk. LWT 101, 404–409. doi: 10.1016/j.lwt.2018.11.047

[ref59] Gentès M. C. Turgeon S. St-Gelais D. (2015). Impact of starch and exopolysaccharide-producing lactic acid bacteria on the properties of set and stirred yoghurts. Int. Dairy J. 55, 79–86. doi: 10.1016/j.idairyj.2015.12.006

[ref60] Gharibzahedi S. M. T. Chronakis I. S. (2018). Crosslinking of milk proteins by microbial transglutaminase: utilization in functional yogurt products. Food Chem. 245, 620–632. doi: 10.1016/j.foodchem.2017.10.138, 29287418

[ref61] Gilbert A Rioux LE St-Gelais D Turgeon S. (2020). Characterization of syneresis phenomena in stirred acid milk gel using low frequency nuclear magnetic resonance on hydrogen and image analyses. Available online at: https://hdl.handle.net/20.500.11794/104103

[ref62] Glantz M. Devold T. G. Vegarud G. E. Lindmark Månsson H. Stålhammar H. Paulsson M. (2010). Importance of casein micelle size and milk composition for milk gelation. J. Dairy Sci. 93, 1444–1451. doi: 10.3168/jds.2009-2856, 20338421

[ref63] González-Olivares L. G. Añorve-Morga J. Castañeda-Ovando A. Contreras-López E. Jaimez-Ordaz J. (2014). Peptide separation of commercial fermented milk during refrigerated storage. Food Sci. Technol 34, 674–679. doi: 10.1590/1678-457x.6415

[ref64] Hailu Y. Hansen E. B. Seifu E. Eshetu M. Ipsen R. Kappeler S. (2016). Functional and technological properties of camel milk proteins: a review. J. Dairy Res. 83, 422–429. doi: 10.1017/s0022029916000686, 27845026

[ref65] Hamed N. S. Mbye M. Ayyash M. Ulusoy B. H. Kamal-Eldin A. (2024). Camel milk: antimicrobial agents, fermented products, and shelf life. Foods 13:381. doi: 10.3390/foods13030381, 38338516 PMC10855775

[ref66] Han M. Liao W. Y. Wu S. M. Gong X. Bai C. (2020). Use of *Streptococcus thermophilus* for the in situ production of γ-aminobutyric acid-enriched fermented milk. J. Dairy Sci. 103, 98–105. doi: 10.3168/jds.2019-16856 31668446

[ref67] Han Y. Zhu L. Zhang H. Liu T. Wu G. (2023). Characteristic of the interaction mechanism between soy protein isolate and functional polysaccharide with different charge characteristics and exploration of the foaming properties. Food Hydrocoll. 150:109615. doi: 10.1016/j.foodhyd.2023.109615

[ref68] Hashim I. B. Khalil A. H. Habib H. (2009). Quality and acceptability of a set-type yogurt made from camel milk. J. Dairy Sci. 92, 857–862. doi: 10.3168/jds.2008-1408, 19233778

[ref69] Hassan A. N. Ipsen R. Janzen T. Qvist K. B. (2003). Microstructure and rheology of yogurt made with cultures differing only in their ability to produce exopolysaccharides. J. Dairy Sci. 86, 1632–1638. doi: 10.3168/jds.S0022-0302(03)73748-5, 12778573

[ref70] Hati S. (2018). Fermented camel milk: a review on its bio-functional properties. Emir. J. Food Agric. 30:268. doi: 10.9755/ejfa.2018.v30.i4.1661

[ref71] Hattem H. E. Manal A. N. Hanna S. S. Elham A. A. (2011). A study on the effect of thermal treatment on composition and some properties of camel milk. Slovak J. Anim. Sci. 44, 97–102.

[ref72] Hayek S. A. Ibrahim S. A. (2013). Current limitations and challenges with lactic acid bacteria: a review. Food Nutr. Sci. 4, 73–87. doi: 10.4236/fns.2013.411A010

[ref73] Ho T. M. Zhao J. Bansal N. (2022a). Acid gelation properties of camel Milk—effect of gelatin and processing conditions. Food Bioprocess Technol. 15, 2363–2373. doi: 10.1007/s11947-022-02890-5

[ref74] Ho T. M. Zou Z. Bansal N. (2022b). Camel milk: a review of its nutritional value, heat stability, and potential food products. Food Res. Int. 153:110870. doi: 10.1016/j.foodres.2021.110870, 35227464

[ref75] Hovjecki M. Miloradovic Z. Mirkovic N. Radulovic A. Pudja P. Miocinovic J. (2021). Rheological and textural properties of goat’s milk set-type yoghurt as affected by heat treatment, transglutaminase addition and storage. J. Sci. Food Agric. 101, 5898–5906. doi: 10.1002/jsfa.11242, 33798268

[ref76] Hu Y. Zhang L. Wen R. Chen Q. Kong B. (2022). Role of lactic acid bacteria in flavor development in traditional Chinese fermented foods: a review. Crit. Rev. Food Sci. Nutr. 62, 2741–2755. doi: 10.1080/10408398.2020.1858269, 33377402

[ref77] Ibrahem S. A. El Zubeir I. E. M. (2016). Processing, composition and sensory characteristic of yoghurt made from camel milk and camel–sheep milk mixtures. Small Rumin. Res. 136, 109–112. doi: 10.1016/j.smallrumres.2016.01.014

[ref78] Ibrahim A. Khalifa S. (2015). Improve sensory quality and textural properties of fermented camel’s Milk by fortified with dietary Fiber. J. Am. Sci. 1111, 42–54.

[ref79] Imamou Hassani M. Saikia D. Walia A. (2022). Nutritional and therapeutic value of camel milk. Int. J. Appl. Res. 8, 01–05. doi: 10.22271/allresearch.2022.v8.i4a.9614

[ref80] Imen F. Ziadi M. Moufida A. Naziha A. Arroum S. Assadi M. . (2015). Isolation and characterisation of lactic acid bacteria strains from raw camel milk for potential use in the production of fermented Tunisian dairy products. Int. J. Dairy Technol. 69, 103–113. doi: 10.1111/1471-0307.12226

[ref81] Izadi A. Khedmat L. Mojtahedi S. Y. (2019). Nutritional and therapeutic perspectives of camel milk and its protein hydrolysates: a review on versatile biofunctional properties. J. Funct. Foods 60:103441. doi: 10.1016/j.jff.2019.103441

[ref82] Jasim A. Salih G. Hamk M. (2018). The effect of Arabic gum on physicochemical and sensory properties of camel milk, yogurt article info abstract. J Zankoy Sulaimani Part A. 1, 97–104. doi: 10.17656/jzs.10656

[ref83] Jrad Z. Oussaief O. Zaidi S. Khorchani T. El-Hatmi H. (2021). Co-fermentation process strongly affect the nutritional, texture, syneresis, fatty acids and aromatic compounds of dromedary UF-yogurt. J. Food Sci. Technol. 58, 1727–1739. doi: 10.1007/s13197-020-04682-1, 33897011 PMC8021673

[ref84] Kamal-Eldin A. Alhammadi A. Gharsallaoui A. Hamed F. Ghnimi S. (2020). Physicochemical, rheological, and micro-structural properties of yogurts produced from mixtures of camel and bovine milks. NFS J. 19, 26–33. doi: 10.1016/j.nfs.2020.05.001

[ref85] Kamal-Eldin A Ayyash M Sobti B Nagy P. Camel milk. (2021). In: Encyclopedia of dairy sciences. Elsevier; 504–513. Available online at: http://www.scopus.com/inward/record.url?scp=85131428364&partnerID=8YFLogxK

[ref86] Kanamarlapudi S. L. R. K. Muddada S. (2017). Characterization of exopolysaccharide produced by *Streptococcus thermophilus* CC30. Biomed. Res. Int. 2017:4201809. doi: 10.1155/2017/4201809, 28815181 PMC5549498

[ref87] Kappeler S. Farah Z. Puhan Z. (1998). Sequence analysis of *Camelus dromedarius* milk caseins. J. Dairy Res. 65, 209–222. doi: 10.1017/s0022029997002847, 9627840

[ref88] Karimi G. Sabran M. R. Jamaluddin R. Parvaneh K. Mohtarrudin N. Ahmad Z. . (2015). The anti-obesity effects of *Lactobacillus casei* strain Shirota versus orlistat on high fat diet-induced obese rats. Food Nutr. Res. 59:29273. doi: 10.3402/fnr.v59.29273, 26699936 PMC4689799

[ref89] Kavas N. (2016). Yoghurt production from camel (Camelus dramedarius) milk fortified with samphire molasses and different colloids. Mljekarstvo Dairy. 66, 34–47. doi: 10.15567/mljekarstvo.2016.0104

[ref90] Kenari R. Razavi R. (2021). Effect of sonication conditions: time, temperature and amplitude on physicochemical, textural and sensory properties of yoghurt. Int. J. Dairy Technol. 74, 332–343. doi: 10.1111/1471-0307.12761

[ref91] Khalesi M. Salami M. Moslehishad M. Winterburn J. Moosavi-Movahedi A. (2017). Biomolecular content of camel milk: a traditional superfood towards future healthcare industry. Trends Food Sci. Technol. 62, 49–58. doi: 10.1016/j.tifs.2017.02.004

[ref92] Khalifa M. Zakaria A. (2018). Physiochemical, sensory characteristics and acceptability of a new set yogurt developed from camel and goat Milk mixed with Buffalo Milk. Adv. Anim. Vet. Sci. 7, 172–177. doi: 10.17582/journal.aavs/2019/7.3.172.177

[ref93] Kieliszek M. Pobiega K. Piwowarek K. Kot A. M. (2021). Characteristics of the proteolytic enzymes produced by lactic acid bacteria. Molecules 26:1858. doi: 10.3390/molecules26071858, 33806095 PMC8037685

[ref94] Kim S. Y. Hyeonbin O. Lee P. Kim Y. S. (2020). The quality characteristics, antioxidant activity, and sensory evaluation of reduced-fat yogurt and nonfat yogurt supplemented with basil seed gum as a fat substitute. J. Dairy Sci. 103, 1324–1336. doi: 10.3168/jds.2019-17117, 31785875

[ref95] Konuspayeva G. Faye B. (2021). Recent advances in camel milk processing. Animals 11:1045. doi: 10.3390/ani11041045, 33917722 PMC8068116

[ref96] Körzendörfer A. Nöbel S. Hinrichs J. (2017). Particle formation induced by sonication during yogurt fermentation—impact of exopolysaccharide-producing starter cultures on physical properties. Food Res. Int. 97, 170–177. doi: 10.1016/j.foodres.2017.04.006, 28578038

[ref97] Kumar D. Verma A. Chatli M. K. Singh R. Kumar P. Mehta N. . (2015). Camel milk: alternative milk for human consumption and its health benefits. Nutr. Food Sci. 46, 217–227. doi: 10.1108/NFS-07-2015-0085

[ref98] Ladjevardi Z. Yarmand M. Emam-Djomeh Z. Niasari-Naslaji A. (2016). Physicochemical properties and viability of probiotic bacteria of functional synbiotic camel yogurt affected by oat β-glucan during storage. J. Agric. Sci. Technol. 18, 1233–1246.

[ref99] Lakemond C. van Vliet T. (2008). Acid skim milk gels: the gelation process as affected by preheating pH. Int. Dairy J. 18, 574–584. doi: 10.1016/j.idairyj.2007.11.001

[ref100] Laneuville S. Turgeon S. (2014). Microstructure and stability of skim milk acid gels containing an anionic bacterial exopolysaccharide and commercial polysaccharides. Int. Dairy J. 37. doi: 10.1016/j.idairyj.2014.01.014

[ref101] Lean I. (2011). Encyclopedia of dairy sciences: 2nd edition. Academic Press (Elsevier). vol. 2, 246–249.

[ref102] Lecomte X. Gagnaire V. Lortal S. Dary A. Genay M. (2016). *Streptococcus thermophilus*, an emerging and promising tool for heterologous expression: advantages and future trends. Food Microbiol. 53, 2–9. doi: 10.1016/j.fm.2015.05.003, 26611164

[ref103] Lee W. J. Lucey J. A. (2004). Structure and physical properties of yogurt gels: effect of inoculation rate and incubation temperature. J. Dairy Sci. 87, 3153–3164. doi: 10.3168/jds.S0022-0302(04)73450-5, 15377593

[ref104] Lee W. J. Lucey J. A. (2010). Formation and physical properties of yogurt. Asian Australas. J. Anim. Sci. 23, 1127–1136. doi: 10.5713/ajas.2010.r.05

[ref105] Lesme H. Rannou C. Famelart M. H. Bouhallab S. Prost C. (2020). Yogurts enriched with milk proteins: texture properties, aroma release and sensory perception. Trends Food Sci. Technol. 98, 140–149. doi: 10.1016/j.tifs.2020.02.006

[ref106] Li C. Song J. Kwok L. Y. Wang J. Dong Y. Yu H. . (2017). Influence of *Lactobacillus plantarum* on yogurt fermentation properties and subsequent changes during postfermentation storage. J. Dairy Sci. 100, 2512–2525. doi: 10.3168/jds.2016-11864, 28215898

[ref107] Li S. Tang S. He Q. Hu J. Zheng J. (2019). Changes in proteolysis in fermented milk produced by *Streptococcus thermophilus* in co-culture with *Lactobacillus plantarum* or *Bifidobacterium animalis* subsp. *lactis* during refrigerated storage. Mol. Basel Switz. 24:3699. doi: 10.3390/molecules24203699 PMC6833003 31618866

[ref108] Liu E. Zheng H. Shi T. Ye L. Konno T. Oda M. . (2016). Relationship between *Lactobacillus bulgaricus* and *Streptococcus thermophilus* under whey conditions: focus on amino acid formation. Int. Dairy J. 56. doi: 10.1016/j.idairyj.2016.01.019

[ref109] Lund A. Shah A. H. Jatoi A. S. Khaskheli G. Malhi M. Khaskheli A. . (2019). Effect of heating on shelf life and sensory characteristics of camel milk. Pure Appl. Biol. 9, 74–79.

[ref110] Ma Y. S. Zhao H. J. Zhao X. H. (2019). Comparison of the effects of the Alcalase-hydrolysates of caseinate, and of fish and bovine gelatins on the acidification and textural features of set-style skimmed yogurt-type products. Foods (Basel, Switz.). 8:501. doi: 10.3390/foods8100501 PMC6835843 31618925

[ref111] Mahmoudi M. Khomeiri M. Saeidi M. Kashaninejad M. Davoodi H. (2019). Study of potential probiotic properties of lactic acid bacteria isolated from raw and traditional fermented camel milk. J. Agric. Sci. Technol. 21, 1161–1172.

[ref112] Mahomud M. S. Haque M. A. Akhter N. Asaduzzaman M. (2021). Effect of milk pH at heating on protein complex formation and ultimate gel properties of free-fat yoghurt. J. Food Sci. Technol. 58, 1969–1978. doi: 10.1007/s13197-020-04708-8, 33897033 PMC8021628

[ref113] Mahomud M. S. Katsuno N. Nishizu T. (2017). Role of whey protein-casein complexes on yoghurt texture. Rev. Agric. Sci. 5, 1–12. doi: 10.7831/ras.5.1

[ref114] Marete P. K. Mariga A. M. Huka G. Musalia L. Marete E. Mathara J. M. . (2024). Camel milk products beyond yoghurt and fresh milk: challenges, processing and applications. J. Food Sci. Technol. 61, 220–229. doi: 10.1007/s13197-022-05664-1, 38196715 PMC10772132

[ref115] Mbye M Ayyash M Abu-Jdayil B Kamal-Eldin A. (2022). The texture of camel milk cheese: effects of milk composition, coagulants, and processing conditions. Front. Nutr. 9. Available online at: https://www.frontiersin.orghttps://www.frontiersin.org/journals/nutrition/articles/10.3389/fnut.2022.868320/full 10.3389/fnut.2022.868320 PMC9062519 35520282

[ref116] Meletharayil G. H. Patel H. A. Huppertz T. (2015). Rheological properties and microstructure of high protein acid gels prepared from reconstituted milk protein concentrate powders of different protein contents. Int. Dairy J. 47, 64–71. doi: 10.1016/j.idairyj.2015.02.005

[ref117] Mende S. Rohm H. Jaros D. (2016). Influence of exopolysaccharides on the structure, texture, stability and sensory properties of yoghurt and related products. Int. Dairy J. 52, 57–71. doi: 10.1016/j.idairyj.2015.08.002

[ref118] Metwalli A. A. Ismail E. A. Elkhadragy M. F. Yehia H. M. (2023). Physicochemical, microbiological, and sensory properties of set-type yoghurt supplemented with camel casein hydrolysate. Fermentation 9:353. doi: 10.3390/fermentation9040353

[ref119] Mishra B. K. Hati S. Das S. Prajapati J. B. (2019). Biofunctional attributes and storage study of soy milk fermented by *Lactobacillus rhamnosus* and *Lactobacillus helveticus*. Food Technol. Biotechnol. 57, 399–407. doi: 10.17113/ftb.57.03.19.6103, 31866753 PMC6902289

[ref120] Mohamed H. Ayyash M. Kamal-Eldin A. (2022b). Effect of heat treatments on camel milk proteins – a review. Int. Dairy J. 133:105404. doi: 10.1016/j.idairyj.2022.105404

[ref121] Mohamed H. Johansson M. Lundh Å. Nagy P. Kamal-Eldin A. (2020). Short communication: caseins and α-lactalbumin content of camel milk (*Camelus dromedarius*) determined by capillary electrophoresis. J. Dairy Sci. 103, 11094–11099. doi: 10.3168/jds.2020-19122, 33069408

[ref122] Mohamed H. Nagy P. Agbaba J. Kamal-Eldin A. (2021). Use of near and mid infra-red spectroscopy for analysis of protein, fat, lactose and total solids in raw cow and camel milk. Food Chem. 334:127436. doi: 10.1016/j.foodchem.2020.127436 32711262

[ref123] Mohamed H. Ranasinghe M. Amir N. Nagy P. Gariballa S. Adem A. . (2022a). A study on variability of bioactive proteins in camel (*Camelus dromedarius*) milk: insulin, insulin-like growth factors, lactoferrin, immunoglobulin G, peptidoglycan recognition protein-1, lysozyme and lactoperoxidase. Int. J. Dairy Technol. 75, 289–297. doi: 10.1111/1471-0307.12836

[ref124] Mohsin A. Ni H. Luo Y. Wei Y. Tian X. Guan W. . (2019). Qualitative improvement of camel milk date yoghurt by addition of biosynthesized xanthan from orange waste. LWT 108, 61–68. doi: 10.1016/j.lwt.2019.03.039

[ref125] Moslehishad M. Ehsani M. Salami M. Mirdamadi S. Ezzatpanah H. Naslaji A. . (2013). The comparative assessment of ACE-inhibitory and antioxidant activities of peptide fractions obtained from fermented camel and bovine milk by *Lactobacillus rhamnosus* PTCC 1637. Int. Dairy J. 29, 82–87. doi: 10.1016/j.idairyj.2012.10.015

[ref126] Mudgil P. Jumah B. Hamed F. Ahmed M. Maqsood S. (2018). Rheological, micro-structural and sensorial properties of camel milk yogurt as influenced by gelatin. LWT 98, 646–653. doi: 10.1016/j.lwt.2018.09.008

[ref127] Mullaicharam A. R. (2014). A review on medicinal properties of camel milk. World J. Pharm. Sci., 237–242.

[ref128] Mustafa E. (2015). The effect of mixing different percentages of cow Milk on the physiochemical characteristics of camel Milk yoghurt and the sensory evaluation of yoghurt. World J pharm pharm Sci. Available online at: https://www.academia.edu/85029093/The_Effect_of_Mixing_Different_Percentages_of_Cow_Milk_on_the_Physiochemical_Characteristics_of_Camel_Milk_Yoghurt_and_the_Sensory_Evaluation_of_Yoghurt

[ref129] Muthukumaran M. S. Mudgil P. Baba W. N. Ayoub M. A. Maqsood S. (2023). A comprehensive review on health benefits, nutritional composition and processed products of camel milk. Food Rev. Int. 39, 3080–3116. doi: 10.1080/87559129.2021.2008953

[ref130] Nassar K. S. Lu J. Pang X. Ragab E. S. Yue Y. Zhang S. . (2020). Rheological and microstructural properties of rennet gel made from caprine milk treated by HP. J. Food Eng. 267:109710. doi: 10.1016/j.jfoodeng.2019.109710

[ref131] Nguyen H. T. H. Schwendel H. Harland D. Day L. (2018). Differences in the yoghurt gel microstructure and physicochemical properties of bovine milk containing A1A1 and A2A2 β-casein phenotypes. Food Res. Int. 112, 217–224. doi: 10.1016/j.foodres.2018.06.043, 30131131

[ref132] O’Kennedy B. T. Mounsey J. S. Murphy F. Duggan E. Kelly P. M. (2006). Factors affecting the acid gelation of sodium caseinate. Int. Dairy J. 16, 1132–1141. doi: 10.1016/j.idairyj.2005.11.003

[ref133] Olaimat A. Tarique M. Al Nabulsi A. Bamigbade G. Ali L. MinAllah S. . (2023). Enhancing the rheological, gelation, and functional properties of camel milk yogurt with pea extract. ACS Food Sci. Technol. 3, 1988–2000. doi: 10.1021/acsfoodscitech.3c00366

[ref134] Omar H. El-Nimer A. Ahmed M. Hassaan H. (2019). Production of functional bio—yogurt made from camel milk, skim milk retentate and fortified with sweet potato powder. Egypt. J. Agric. Res. 97, 441–457.

[ref135] Oselu S. Ebere R. Arimi J. M. (2022a). Camels, camel Milk, and camel Milk product situation in Kenya in relation to the world. Int. J. Food Sci. 2022:1237423. doi: 10.1155/2022/1237423 35299617 PMC8923781

[ref136] Oselu S. Ebere R. Huka G. Musalia L. Marete E. Mathara J. M. . (2022b). Production and characterisation of camel milk yoghurt containing different types of stabilising agents. Heliyon 8:e11816. doi: 10.1016/j.heliyon.2022.e11816, 36468136 PMC9708823

[ref137] Park Y. W. Haenlein G. F. W. (2013). Milk and dairy products in human nutrition: Production, composition, and health. Chichester, West Sussex, UK: Wiley-Blackwell.

[ref138] Patel D. Pinto S. Pal M. (2022). A comprehensive review on the properties of camel milk and milk products 6, 200–207. doi: 10.26855/ijfsa.2022.06.010

[ref139] Permyakov E. A. (2020). α-lactalbumin, amazing calcium-binding protein. Biomolecules 10:1210. doi: 10.3390/biom10091210, 32825311 PMC7565966

[ref140] Prasanna P. H. P. Ranadheera S. Vidanarachchi J. (2018). Microstructural aspects of yogurt and fermented Milk. Wiley. 181–208.

[ref141] Puvanenthiran A. Williams R. P. W. Augustin M. A. (2002). Structure and visco-elastic properties of set yoghurt with altered casein to whey protein ratios. Int. Dairy J. 12, 383–391. doi: 10.1016/s0958-6946(02)00033-x

[ref142] Ragab E. Yacoub S. Nassar K. Zhang S. Lv J. (2021). Textural and microstructural properties of set yoghurt produced from goat milk treated by homogenization and thermosonication. Alexandria Sci. Exch. J. 42, 985–995. doi: 10.21608/asejaiqjsae.2021.211545

[ref143] Ramasubramanian L. Restuccia C. Deeth H. C. (2008). Effect of calcium on the physical properties of stirred probiotic yogurt. J. Dairy Sci. 91, 4164–4175. doi: 10.3168/jds.2008-1354, 18946120

[ref144] Raynes J. K. Day L. Augustin M. A. Carver J. A. (2015). Structural differences between bovine a(1) and a(2) β-casein alter micelle self-assembly and influence molecular chaperone activity. J. Dairy Sci. 98, 2172–2182. doi: 10.3168/jds.2014-8800, 25648798

[ref145] Riaz Rajoka M. S. Wu Y. Mehwish H. M. Bansal M. Zhao L. (2020). *Lactobacillus* exopolysaccharides: new perspectives on engineering strategies, physiochemical functions, and immunomodulatory effects on host health. Trends Food Sci. Technol. 103. doi: 10.1016/j.tifs.2020.06.003

[ref146] Rolfe M. D. Rice C. J. Lucchini S. Pin C. Thompson A. Cameron A. D. S. . (2012). Lag phase is a distinct growth phase that prepares bacteria for exponential growth and involves transient metal accumulation. J. Bacteriol. 194, 686–701. doi: 10.1128/jb.06112-11, 22139505 PMC3264077

[ref147] Ryskaliyeva A. Henry C. Miranda G. Faye B. Konuspayeva G. Martin P. (2018). Combining different proteomic approaches to resolve complexity of the milk protein fraction of dromedary, Bactrian camels and hybrids, from different regions of Kazakhstan. PLoS One 13:e0197026. doi: 10.1371/journal.pone.0197026, 29746547 PMC5944991

[ref148] Saleh M. Al-Baz F. Al-Ismail K. (2018). Effects of hydrocolloids as fat replacers on the physicochemical properties of produced Labneh. J. Texture Stud. 49, 113–120. doi: 10.1111/jtxs.12296, 28836674

[ref149] Salih M. Hamid O. (2013). Effect of fortifying camel’s Milk with skim Milk powder on the physicochemical, microbiological and sensory characteristics of set yoghurt. Adv. J. Food Sci. Technol. 5, 765–770. doi: 10.19026/ajfst.5.3161

[ref150] Sasaki Y. Horiuchi H. Kawashima H. Mukai T. Yamamoto Y. (2014). NADH oxidase of *Streptococcus thermophilus* 1131 is required for the effective yogurt fermentation with *Lactobacillus delbrueckii* subsp. *bulgaricus* 2038. Biosci. Microbiota Food Health 33, 31–40. doi: 10.12938/bmfh.33.31, 24936380 PMC4034325

[ref151] Seifu E. (2023). Camel milk products: innovations, limitations and opportunities. Food Prod. Process. Nutr. 5:15. doi: 10.1186/s43014-023-00130-7

[ref152] Seyiti S. Kelimu A. Yusufu G. (2024). Bactrian camel milk: chemical composition, bioactivities, processing techniques, and economic potential in China. Molecules 29:4680. doi: 10.3390/molecules29194680, 39407609 PMC11478162

[ref153] Shi M. Ahtesh F. Mathai M. Mcainch A. Su X. (2016). Effects of fermentation conditions on the potential anti-hypertensive peptides released from yogurt fermented by *Lactobacillus helveticus* and Flavourzyme^®^. Int. J. Food Sci. Technol. 52, 137–145. doi: 10.1111/ijfs.13253

[ref154] Shori A. B. (2017). Camel milk and its fermented products as a source of potential probiotic strains and novel food cultures: a mini review. PharmaNutrition 5, 84–88. doi: 10.1016/j.phanu.2017.06.003

[ref155] Sieuwerts S. (2016). Microbial interactions in the yoghurt consortium: current status and product implications. SOJ Microbiol. Infect. Dis. 4, 01–05. doi: 10.15226/sojmid/4/2/00150

[ref156] Sinaga H. Bansal N. Bhandari B. (2016). Effects of milk pH alteration on casein micelle size and gelation properties of milk. Int. J. Food Prop. 20, 179–197. doi: 10.1080/10942912.2016.1152480

[ref157] Singh R. Mal G. Kumar D. Patil N. V. Pathak K. M. L. (2017). Camel milk: an important natural adjuvant. Agric. Res. 6, 327–340. doi: 10.1007/s40003-017-0284-4

[ref158] Smid E. J. Kleerebezem M. (2014). Production of aroma compounds in lactic fermentations. Annu. Rev. Food Sci. Technol. 5, 313–326. doi: 10.1146/annurev-food-030713-092339, 24580073

[ref159] Smiddy M. A. Huppertz T. van Ruth S. M. (2012). Triacylglycerol and melting profiles of milk fat from several species. Int. Dairy J. 24, 64–69. doi: 10.1016/j.idairyj.2011.07.001

[ref160] Sobti B. Alhefeiti R. M. S. Alahdali F. A. Al Samri M. A. M. Kamal-Eldin A. (2023). Supplementation of drinkable yogurt (Laban) from camel milk with fruit purees improves its quality and sensory properties. NFS J. 32:100143. doi: 10.1016/j.nfs.2023.100143

[ref161] Sobti B. Aljneibi A. H. A. Seraidy H. A. A. Alnaqbi A. A. H. Al Zain B. Ramachandran T. . (2021). Short communication: the effect of pectin and sodium alginate on labans made from camel milk and bovine milk. J. Dairy Sci. 104, 5279–5284. doi: 10.3168/jds.2020-19220, 33663820

[ref162] Sobti B. Ayyash M. Mbye M. Ranasinghe M. Nazir A. Kamleh R. . (2024). Effects of ultrafiltration followed by heat or high-pressure treatment on camel and bovine yogurts. NFS J. 35:100181. doi: 10.1016/j.nfs.2024.100181

[ref163] Sobti B. Kamal-Eldin A. (2019). Effect of added bovine casein and whey protein on the quality of camel and bovine Milk yoghurts. Emir. J. Food Agric., 804–811. doi: 10.9755/ejfa.2019.v31.i10.2022

[ref164] Sobti B. Mbye M. Alketbi H. Alnaqbi A. Alshamisi A. Almeheiri M. . (2020). Rheological characteristics and consumer acceptance of camel milk yogurts as affected by bovine proteins and hydrocolloids. Int. J. Food Prop. 23, 1347–1360. doi: 10.1080/10942912.2020.1797785

[ref165] Soleymanzadeh N. Mirdamadi S. Kianirad M. (2016). Antioxidant activity of camel and bovine milk fermented by lactic acid bacteria isolated from traditional fermented camel milk (Chal). Dairy Sci. Technol. 96, 443–457. doi: 10.1007/s13594-016-0278-1

[ref166] Swelum A. A. El-Saadony M. T. Abdo M. Ombarak R. A. Hussein E. O. S. Suliman G. . (2021). Nutritional, antimicrobial and medicinal properties of camel’s milk: a review. Saudi J. Biol. Sci. 28, 3126–3136. doi: 10.1016/j.sjbs.2021.02.057, 34025186 PMC8117040

[ref167] Tadesse S. A. Emire S. A. (2020). Production and processing of antioxidant bioactive peptides: a driving force for the functional food market. Heliyon 6:e04765. doi: 10.1016/j.heliyon.2020.e04765, 32913907 PMC7472861

[ref168] Tarrah A. Treu L. Giaretta S. Duarte V. Corich V. Giacomini A. (2018). Differences in carbohydrates utilization and antibiotic resistance between *Streptococcus macedonicus* and *Streptococcus thermophilus* strains isolated from dairy products in Italy. Curr. Microbiol. 75, 1334–1344. doi: 10.1007/s00284-018-1528-7, 29916034

[ref169] Tavakoli M. Habibi Najafi M. B. Mohebbi M. (2019). Effect of the milk fat content and starter culture selection on proteolysis and antioxidant activity of probiotic yogurt. Heliyon. 5:e01204. doi: 10.1016/j.heliyon.2019.e01204, 30766933 PMC6360988

[ref170] Tiwari S. Kavitake D. Devi P. B. Halady Shetty P. (2021). Bacterial exopolysaccharides for improvement of technological, functional and rheological properties of yoghurt. Int. J. Biol. Macromol. 183, 1585–1595. doi: 10.1016/j.ijbiomac.2021.05.140, 34044028

[ref171] Tomotake H. Okuyama R. Katagiri M. Fuzita M. Yamato M. Ota F. (2006). Comparison between Holstein cow’s milk and Japanese-Saanen goat’s milk in fatty acid composition, lipid digestibility and protein profile. Biosci. Biotechnol. Biochem. 70, 2771–2774. doi: 10.1271/bbb.60267, 17090948

[ref172] Toshimitsu T. Mochizuki J. Ikegami S. Itou H. (2016). Identification of a *Lactobacillus plantarum* strain that ameliorates chronic inflammation and metabolic disorders in obese and type 2 diabetic mice. J. Dairy Sci. 99, 933–946. doi: 10.3168/jds.2015-9916 26686731

[ref173] Trujillo A. J. Roig-Sagués A. Zamora A. Ferragut V. (2016). High-pressure homogenization for structure modification. Woodhead Publishing. 315–344.

[ref174] Tsermoula P. Khakimov B. Nielsen J. Engelsen S. (2021). Whey—the waste-stream that became more valuable than the food product. Trends Food Sci. Technol. 118, 230–241. doi: 10.1016/j.tifs.2021.08.025

[ref175] Wang J. Li J. Luo L. Chen Z. Mao L. Wang F. . (2025). New insights into the acid gelation of camel milk casein. J. Agric. Food Res. 19:101746. doi: 10.1016/j.jafr.2025.101746

[ref176] Wang D. Wusigale n. Li L. Bai L. Chen Y. (2025). Effects of Lacticaseibacillus casei Zhang addition on physicochemical properties and metabolomics of fermented camel milk during storage. Food Chem. X 26:102318. doi: 10.1016/j.fochx.2025.102318 40092409 PMC11910121

[ref177] Wang J. Zhao X. Tian Z. Yang Y. Yang Z. (2015). Characterization of an exopolysaccharide produced by *Lactobacillus plantarum* YW11 isolated from Tibet kefir. Carbohydr. Polym. 125, 16–25. doi: 10.1016/j.carbpol.2015.03.003, 25857955

[ref178] Wu H. Hulbert G. Mount J. (2000). Effects of ultrasound on milk homogenization and fermentation with yogurt starter. Innov. Food Sci. Emerg. Technol. 1, 211–218. doi: 10.1016/S1466-8564(00)00020-5

[ref179] Xu Y. Cui Y. Wang X. Yue F. Shan Y. Liu B. . (2019). Purification, characterization and bioactivity of exopolysaccharides produced by *Lactobacillus plantarum* KX041. Int. J. Biol. Macromol. 128, 480–492. doi: 10.1016/j.ijbiomac.2019.01.117 30682478

[ref180] Xu Z. Guo Q. Zhang H. Wu Y. Hang X. Ai L. (2018). Exopolysaccharide produced by Streptococcus thermophiles S-3: molecular, partial structural and rheological properties. Carbohydr. Polym. 194, 132–138. doi: 10.1016/j.carbpol.2018.04.014, 29801820

[ref181] Yang S. Bai M. Kwok L. Y. Zhong Z. Sun Z. (2025). The intricate symbiotic relationship between lactic acid bacterial starters in the milk fermentation ecosystem. Crit. Rev. Food Sci. Nutr. 65, 728–745. doi: 10.1080/10408398.2023.2280706, 37983125

[ref182] Yang T. Wu K. Wang F. Liang X. Liu q. Li G. . (2014). Effect of exopolysaccharides from lactic acid bacteria on the texture and microstructure of buffalo yoghurt. Int. Dairy J. 34, 252–256. doi: 10.1016/j.idairyj.2013.08.007

[ref183] Yirda A. Guya M. Woldeyes K. (2020). Current status of camel dairy processing and technologies: a review. Open J. Anim. Sci. 10, 362–377. doi: 10.4236/ojas.2020.103022

[ref184] Yu P. Li N. Geng M. Liu Z. Liu X. Zhang H. . (2020). Short communication: lactose utilization of *Streptococcus thermophilus* and correlations with β-galactosidase and urease. J. Dairy Sci. 103, 166–171. doi: 10.3168/jds.2019-17009, 31704010

[ref185] Zaręba D. Ziarno M. Scibisz I. Gawron J. (2014). The importance of volatile compound profile in the assessment of fermentation conducted by *Lactobacillus casei* DN-114 001. Int. Dairy J. 35, 11–14. doi: 10.1016/j.idairyj.2013.09.009

[ref186] Zhao L. Xie Q. Shi F. Liang S. Chen Q. Evivie S. E. . (2021). Proteolytic activities of combined fermentation with *Lactobacillus helveticus* KLDS 1.8701 and *Lactobacillus plantarum* KLDS 1.0386 reduce antigenic response to cow milk proteins. J. Dairy Sci. 104, 11499–11508. doi: 10.3168/jds.2021-20668, 34454765

[ref187] Zhou T. Huo R. Kwok L. Y. Li C. Ma Y. Mi Z. . (2019). Effects of applying *Lactobacillus helveticus* H9 as adjunct starter culture in yogurt fermentation and storage. J. Dairy Sci. 102, 223–235. doi: 10.3168/jds.2018-14602 30343912

