# Supplementary figures and images for: Processing difficulties and biochemical barriers in camel milk fermentation
Source: Front Microbiol. 2026 Feb 2;17:1752671. doi: 10.3389/fmicb.2026.1752671 (PMC12907412; doi:10.3389/fmicb.2026.1752671)

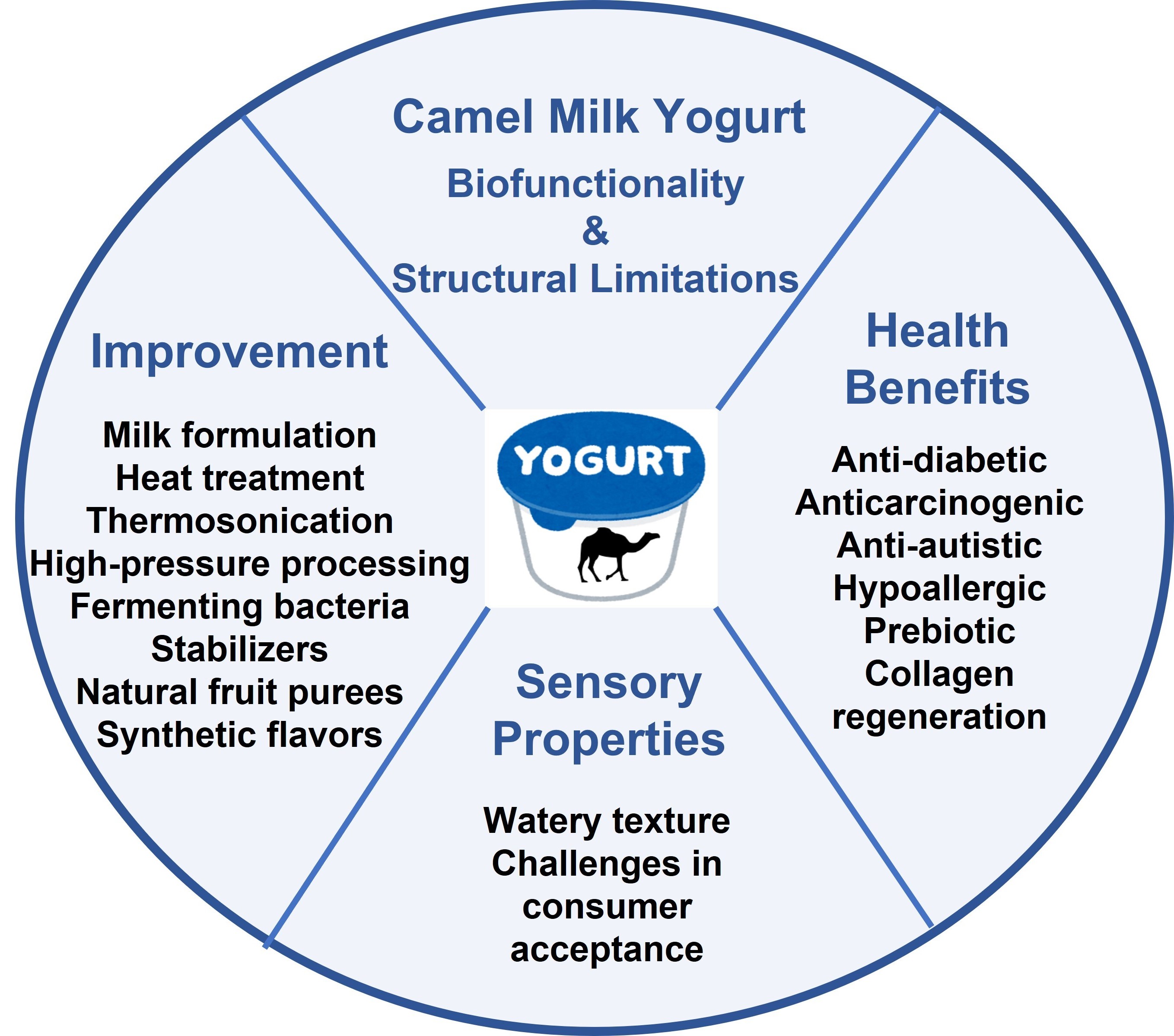

Supplement: Supplementary file 1 [file Image_1.jpeg]
